# Broad Redox Density of States and S–O Functionalities Drive Stable Pseudocapacitive Behavior in Sulfurized Polyacrylonitrile (SPAN) Cathodes

**DOI:** 10.1002/advs.202511459

**Published:** 2025-09-29

**Authors:** Sajib Kumar Mohonta, Nawraj Sapkota, Ramakrishna Podila

**Affiliations:** ^1^ Department of Physics and Astronomy Clemson University Clemson SC 29634 USA

**Keywords:** electrochemistry, energy storage, Li–S batteries, quantum capacitance, redox density of states

## Abstract

Sulfurized polyacrylonitrile (SPAN) cathodes offer a promising route for improving Li–S batteries by eliminating polysulfide shuttling and enabling stable, high‐rate performance. Here, a comprehensive mechanistic study of SPAN cathodes with varying sulfur content (0–35 wt.%), revealing how structural and electronic factors that govern charge storage is presented. Cyclic voltammetry shows that SPAN exhibits distinct redox features without soluble polysulfides, and that higher sulfur content leads to sharper redox peaks and increased capacity. In situ Raman spectroscopy reveals that electrochemical cycling induces the formation of nanocrystalline *sp* ^2^ carbon domains and a decline in φ‐S_*x*_ species. X‐ray photoelectron spectroscopy shows the presence of stable S– O functionalities, including sulfone and sulfonate groups, which are previously unreported in SPAN. These S–O motifs evolve with cycling and are correlated with SPAN's redox activity. Trasatti analysis demonstrates that SPAN's charge storage is dominated by surface‐controlled (pseudocapacitive) processes, unlike the diffusion‐limited (redox) behavior of elemental sulfur. The pseudocapacitive contribution to the total capacity is found to increase with increasing S content. The redox density of states, *g*_*r*_(μ), is further quantified using electrochemical capacitance spectroscopy through a density functional theory (DFT) inspired approach. The broad and stable *g*_*r*_(μ), enabled by diverse S–O redox sites and the active participation of the carbon backbone, underpins SPAN's pseudocapacitive behavior and superior cycling stability.

## Graphical abstract

We report a fundamental mechanism underlying charge storage in sulfurized polyacrylonitrile cathodes. Stable sulfuroxygen groups and a broad redox density of states enable pseudocapacitive behavior distinct from conventional sulfur cathodes. Through combined spectroscopic and electrochemical analysis, this study reveals key structurefunction relationships that advance the design of high‐performance polymer‐based cathodes for lithiumsulfur energy storage systems.

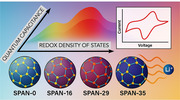

## 1 Introduction

The increasing demand for high‐performance energy storage systems has driven extensive research into next‐generation battery technologies. Among these, Li‐S batteries (LSBs) have garnered significant attention due to their high theoretical energy density of 2600 Wh kg^−1^ and the easy availability and low cost of elemental sulfur (S_8_).^[^ ^1^, ^2^, ^3^, ^4^, ^5^, ^6^, ^7^, ^8^, ^9^, ^10^ ^]^ Despite these advantages, the practical application of LSBs is hindered by several challenges, notably the poor cycling stability and low Coulombic efficiency of S_8_ cathodes caused by the dissolution and shuttling of lithium polysulfides or LiPS (Li_2_S_*n*_, 3 ⩽ *n* ⩽ 8) during charge‐discharge cycles.^[^ ^11^, ^12^, ^13^ ^]^

To address these issues, sulfurized polyacrylonitrile (SPAN) has emerged as a promising alternative cathode material. SPAN, first reported by Wang et al.,^[^ ^14^ ^]^ involves the chemical binding of S within a pyrolyzed polyacrylonitrile (PAN) matrix. The chemical structure of SPAN is often characterized by the presence of short S chains (–S_*x*_–) covalently bonded to the dehydrogenated carbonized backbone through C–S and S‐S bonds.^[^ ^2^, ^14^, ^15^, ^16^, ^17^, ^18^, ^19^, ^20^, ^21^, ^22^, ^23^, ^24^ ^]^ Such short S chains in SPAN have been proposed to help stabilize the structure and prevent the formation of long‐chain LiPS.^[^ ^1^, ^19^ ^]^ The stable chemical environment provided by the carbon backbone in SPAN significantly improves the cycling stability and Coulombic efficiency of LSBs. SPAN exhibits high compatibility with carbonate‐based electrolytes, further enhancing its practical applicability.^[^ ^15^, ^25^, ^26^ ^]^ Additionally, SPAN cathodes are compatible existing lithium‐ion battery electrode coating technology, making it a commercially viable option.

Electrochemical studies suggest that during the discharge process, C–S and S–S bonds in SPAN cleave to form Li_2_S, which is further evidenced by the absence of polysulfide peaks in cyclic voltammetry (CV) and in situ Raman spectra during the lithiation process.^[^ ^15^, ^16^ ^]^ The redox peaks observed in SPAN during CV are distinct from those of S_8_. Despite much progress in SPAN cathodes, it is not yet clear what specific chemical reactions correspond to these processes and how they evolve based on the amount of S in SPAN. Current literature suggests that the Li storage mechanism in SPAN involves reactions not only between S and Li but also with C = N and C = C groups in the carbon backbone.^[^ ^2^, ^14^, ^15^, ^16^, ^17^, ^18^, ^19^, ^20^ ^]^ These interactions have been proposed to form Li‐C‐N‐Li and Li‐C‐C‐Li bonds, contributing to a stable practical discharge capacity.^[^ ^11^ ^]^ However, there is no consensus on the detailed mechanisms of these reactions. Some studies propose that C‐S and S‐S bonds break during the first discharge to form Li_2_S, which then participates in reversible reactions with the carbon backbone. Others suggest different pathways involving the formation and dissolution of various S species and Li compounds.^[^ ^1^, ^12^, ^13^ ^]^ Further studies have utilized advanced characterization techniques such as solid‐state nuclear magnetic resonance (NMR), X‐ray photoelectron spectroscopy (XPS), in situ Raman, and in situ X‐ray absorption spectroscopy (XAS) to investigate the redox mechanisms of SPAN.^[^ ^13^, ^15^, ^16^, ^27^ ^]^ These studies highlight the complexity of the electrochemical reactions and suggest that multiple reaction pathways may coexist, contributing to the overall performance of SPAN cathodes. Interestingly, O atoms are rarely included in the structural description of SPAN although the elemental decomposition of SPAN shows 1–10 wt. % O content.

In our prior work,^[^ ^15^, ^28^ ^]^ we demonstrated the practical viability of SPAN cathodes by fabricating industrial‐grade pouch cells with 35–42 wt.% S and S loadings ⩾ 5 mg cm^−2^. These cells exhibited specific capacities exceeding 1300 mAh $g_{S}^{-1}$ and retained performance over 2400 cycles at 7.5 C with minimal degradation. We showed that high cell‐level energy densities >300 Wh/Kg can be achieved with SPAN at a high S loading (⩾ 5 mg cm^−2^) by optimizing electrolyte‐to‐sulfur (E/S) and negative‐to‐positive (N/P) ratios.^[^ ^15^ ^]^ While S_8_ cathodes offer theoretically higher energy densities compared to SPAN or Li‐ion, their practical deployment is severely hindered by polysulfide shuttling and stability issues. In contrast, SPAN may not match the theoretical energy of S_8_‐based systems, but it offers a simpler, more cost‐effective, and chemically stable alternative that is well‐positioned to surpass conventional Li‐ion batteries in real‐world applications.

In the present study, we move beyond performance metrics to investigate the fundamental mechanisms that govern charge storage in SPAN as a function of S content. We synthesized SPAN samples with S content ranging from 0 to 35 wt.% and characterized them using scan‐rate‐dependent CV, in situ Raman, XPS, and electrochemical capacitance spectroscopy (ECS). Our results revealed that SPAN exhibits fundamentally distinct electrochemical behavior compared to S_8_, driven by both its chemically bound S and an active carbon backbone. We observed that increasing S content enhances pseudocapacitive charge storage, with high S SPAN samples displaying sharper redox features and greater surface‐controlled capacity contributions. Using in situ Raman spectroscopy, we tracked the structural evolution of SPAN during cycling and identified the formation of nanocrystalline *sp* ^2^ C domains, alongside the progressive transformation of S bonding environments. XPS measurements uncovered the presence of stable S– O functionalities, which emerge during cycling and have not been previously incorporated into SPAN structural models. We employed ECS in a density functional theory (DFT)‐inspired framework to extract the redox density of states, *g*_*r*_(μ). We found that SPAN exhibits a broad and stable *g*_*r*_(μ), which underpins its enhanced rate performance and cycling stability. In summary, the novelty of this work lies in: i) the identification of redox‐active S–O functionalities not previously included in SPAN structural models; ii) the use of ECS to extract an experimental redox density of states *g*_*r*_(μ) in a DFT‐like framework; and iii) a quantitative electrochemical dissection of charge storage modes with clear distinction between surface‐ and diffusion‐controlled processes. These findings establish foundational principles for tuning SPAN's structure–function relationships and open new pathways for the rational design of hybrid redox polymers with tailored pseudocapacitance and rate performance.

## 2 Experimental Section

### 2.1 Chemicals and Materials

: Poly (vinylidene fluoride) (PVDF), lithium bis (trifluoromethane) sulfonamide (LiTFSI), ethylene carbonate (EC), 1,3‐dioxolane (DOL), N‐methyl‐2‐pyrrolidone (NMP), polyacrylonitrile (PAN), and 1,2‐ dimethoxy ethane (DME) were purchased from Sigma–Aldrich. Sulfur (S_8_) powder (325 mesh) was purchased from Alfa Aesar. Carbon Super P, Li chips, and carbon‐coated Al foil (thickness 18 µm) were purchased from MTI Corporation (mtixtl.com). Celgard 2325, 25 µm trilayer microporous membrane (Polypropylene (PP), Polyethylene (PE), and Polypropylene (PP)) was purchased from Celgard, LLC.

### 2.2 Sulfurized Polyacrylonitrile (SPAN) Preparation

: PAN and S were mixed in different weight ratios (3:1, 4:1, 5:1, and 6:1) to prepare SPAN samples with different S content. Upon mixing, the powders were loaded into a ceramic boat and annealed in a quartz tube (1 inch diameter) at 450 °C for 6 h at a ramp rate of 5 °C min^−1^ in N_2_ atmosphere. The obtained samples were ball milled for 40 min with two 20‐min cycles and a 10‐min break.

### 2.3 Characterization

: Elemental analysis of C, H, S, N, and O was performed by Atlantic Microlabs (Norcross, GA). The CHNSO analysis of all SPAN samples is provided in **Table** **1**. XPS was performed with Kratos Axis Supra XPS (X‐ray source: monochromated Al K_α_, multichannel plate, and delay line detector with a take‐off angle of 90°). Nitrogen adsorption–desorption isotherms were collected at 77 K using a Quantachrome Autosorb iQ analyzer. Prior to measurement, the sample was degassed at 410 K under vacuum for ≈7 h to remove physisorbed impurities. The Brunauer–Emmett–Teller (BET) surface area was calculated using a multi‐point BET model in the relative pressure range of 0.05–0.30. Pore size distribution and cumulative pore volume were determined from the desorption branch using the Barrett–Joyner–Halenda (BJH) method. in situ micro‐Raman spectra were collected using Renishaw InVia setup equipped with a 532 nm laser. X‐ray diffraction (XRD) was performed using Rigaku SmartLab Powder X‐ray Diffractometer. All samples were characterized using thermogravimetric (TGA) analysis (TA instrument SDT Q600) under nitrogen gas flow at 100 mL min^−1^ from room temperature to 600 °C with a ramp of 20 °C min^−1^. Dynamic light scattering (DLS) was performed using Malvern Zetasizer 90 on SPAN samples suspended in NMP.

**Table 1:** CHSNO analysis of SPAN samples.

Samples	C	H	N	S	O
SPAN‐16	54.50	1.41	18.97	16.66	8.46
SPAN‐19	55.07	1.66	19.37	19.08	4.82
SPAN‐29	48.47	0.85	17.12	29.09	4.47
SPAN‐35	43.33	0.65	16.12	35.01	4.89

Five different samples (SPAN‐0, 16, 19, 29 and 35), where the number indicates the S content (0–35 wt.%), were prepared to investigate the nature of various redox peaks. BET surface area measurements showed that the samples have an average surface area of 23.6 ± 5.4 m^2^ g^−1^, a pore volume of 0.09 ± 0.03 cc g^−1^, and an average pore size of 44.5 ± 1 Å, indicating no major differences in the samples' porosity. TGA and XRD of all the samples are presented in Figures S1 and S2 (Supporting Information). As shown in Figure S1 (Supporting Information), all samples exhibited a multi‐step decomposition profile, with a distinct low‐temperature weight loss near 170–185 °C, corresponding to the loss of S from SPAN matrix. A second minor transition ≈380–395 °C is attributed to structural rearrangements within the polymer matrix, while the major decomposition event occurs at ≈790–795 °C, consistent with the breakdown of the C–S framework. Derivative weight loss curves reveal that all samples exhibit sharp decomposition peaks with the total derivative weight % at ≈790–795 °C increasing as a function of the total S content, which was determined through CHSNO analysis (Table 1). XRD of all samples (Figure S2, Supporting Information) confirms the predominantly amorphous character of SPAN samples with a broad halo around 2θ ≈ 25°, typical of turbostratic carbon. No sharp crystalline S peaks are observed, indicating successful S incorporation into the carbon matrix without phase segregation. DLS analysis (Figure S3, Supporting Information) further supports the consistency of the synthesized powders. All SPAN samples exhibit narrow particle size distributions centered ≈400–450 nm, with no significant aggregation or bimodality. This monodispersity suggests that the ball‐milling and thermal synthesis protocols yield well‐dispersed powders with comparable morphological characteristics, independent of S content.

### 2.4 Coin Cell Preparation and Electrochemical Testing

: Slurry was prepared by stirring SPAN and Super P with PVDF (weight ratio of 70:15:15) in NMP. For electrodes, the slurry was then cast on carbon‐coated Al foil and coated using a doctor blade with a vacuum bed (MTI Corp). The coated electrodes were dried in a vacuum oven at 100 °C overnight. Coupons of 12 mm diameter were punched out using puncher machine (ACEY puncher) and used as the electrode for CR2032 type coin cells. A Li chip (15.6 mm diameter × 0.45 mm thickness) was used as the counter electrode. Celgard 2325 was used as the separator and 1.0 M LiTFSI in EC_0.5_DME_0.25_DOL_0.25_ as the electrolyte. This electrolyte combination was chosen based on the systematic investigation of mixed ether‐carbonate electrolytes as discussed in ref. [28]. The cells were assembled inside an argon (Ar) filled glovebox with H_2_O < 0.5 ppm and O_2_ < 0.5 ppm. All CV and EIS or ECS experiments were carried out using a Gamry Reference 3000 potentiostat. CV was performed at different scan rates from 0.1 to 2 mV s^−1^ in the potential range of 1–3 V. The electrochemical impedance or capacitive spectroscopy (EIS or ECS) measurements were carried out in the frequency range of 100 kHz to 0.1 Hz by applying ac amplitude of 10 mV. The EIS/ECS measurements were performed at different voltages (viz., 3.0, 2.6, 2.4, 2.2, 2.0, 1.8, 1.6, 1.4 and 1.0 V) after initial discharge, 1st, 10th, 50th, and 100th cycles. Equivalent circuit fitting was performed using Gamry Echem Analyst 2.0. The charge/discharge is carried out at 0.5–4 C (with 1C = 1675 mA g^−1^). For XPS measurements, each cell (SPAN‐16, 19, 29, 35 and S_8_) was opened inside a glovebox after initial discharge, 1st, 10th, and 100th cycle. The SPAN or S_8_ electrodes were separated from the cells and rinsed in DOL solution for 30 s and dried inside the glovebox. DOL has relatively lower solubility for polysulfides and does not dissolve the chemically bound sulfur species or interfacial decomposition products that comprise the solid‐electrolyte interface. Therefore, rinsing with DOL ensures removal of physically adsorbed species without compromising the interfacial chemistry probed by XPS.^[^ ^29^, ^30^, ^31^ ^]^ Upon drying, each electrode was placed into a sealed vial and stored in the glove box until the XPS measurements were performed.

All the electrochemical and spectroscopic data shown in this article are taken as triplicates *n* = 3 with at least three different cells for each sample to ensure appropriate statistical averaging. All CV peak deconvolutions were performed using Igor Pro v8.2 (Wavemetrics), utilizing the multi‐peak fitting module. Each peak was fit using a Gaussian function, and the fitting parameters–such as peak position, full width at half maximum (FWHM), and area–were obtained from the software's output with an R^2^ value of greater than 0.995 for all fits. The initial estimates for peak positions and potential ranges were obtained using Gamry Echem Analyst 2.0, which was used to extract and visualize the raw CV data and its first derivative. These derivatives were used to identify inflection points that served as initial estimates for peak centers. The number of peaks in each spectrum was determined based on visual inspection of inflection points in the raw CV curve and corroborated by the first derivative to avoid overfitting.

## 3 Results and Discussion

**Figure 1:**
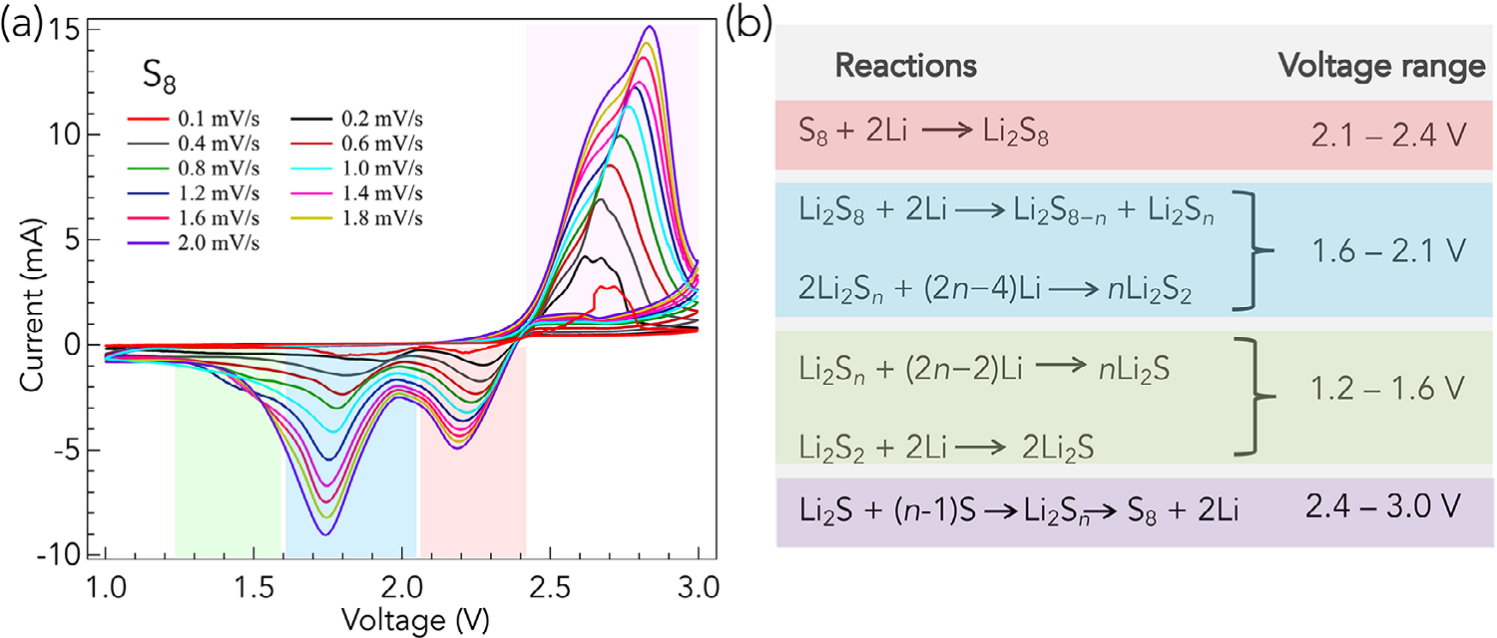
a) Cyclic voltammetry curves of elemental sulfur cathodes measured at various scan rates, illustrating the multi‐step electrochemical reduction and oxidation processes characteristic of S_8_. Distinct cathodic peaks are observed between 2.1–2.4 V (shaded in orange; corresponding to the formation of Li_2_S_8_), 1.6–2.1 V (shaded in blue; related to the formation of long‐chain Li_2_S_*n*_, 4 ⩽ *n* ⩽ 8), and 1.2–1.6 V (shaded in green; arises from the formation of Li_2_S). A broad anodic peak in the 2.4–3.0 V (shaded in purple) range reflects the reverse oxidation processes. b) Schematic representation of the stepwise redox reactions and their corresponding voltage ranges. Reactions are color‐coded to match the shaded voltage windows shown in Figure 1a. During discharge, S_8_ is reduced to Li_2_S through intermediate soluble polysulfides (Li_2_S_n_), which can diffuse through the electrolyte and degrade battery performance via a dissolution–shuttle mechanism. Upon charging, Li_2_S is oxidized back to S_8_ through successive stages.

In elemental sulfur cathodes, S_8_ undergoes a multi‐step electrochemical reduction. As shown in **Figure** **1**a, CV curves for S_8_ exhibit multiple peaks at distinct voltages corresponding to the reactions depicted in Figure 1b. Initially, S_8_ is reduced to Li_2_S_8_ in the 2.1–2.4 V range *vs*. Li/Li^+^, followed by its conversion into long‐chain soluble Li_2_S_*n*_ species (4 ⩽ *n* ⩽ 8), typically observed between 1.6 and 2.1 V. These soluble species can diffuse through the electrolyte and react with the Li anode, resulting in active material loss and degradation of battery performance. Further reduction leads to the formation of Li_2_S in the 1.2–1.6 V range.^[^ ^32^ ^]^ Upon charging, the oxidation follows the reverse sequence: Li_2_S is oxidized to short‐chain Li_2_S_*n*_, which are subsequently converted to longer‐chain Li_2_S_*n*_, and finally to S_8_, with a broad oxidation peak appearing in the 2.4–3.0 V range.^[^ ^32^ ^]^

**Figure 2:**
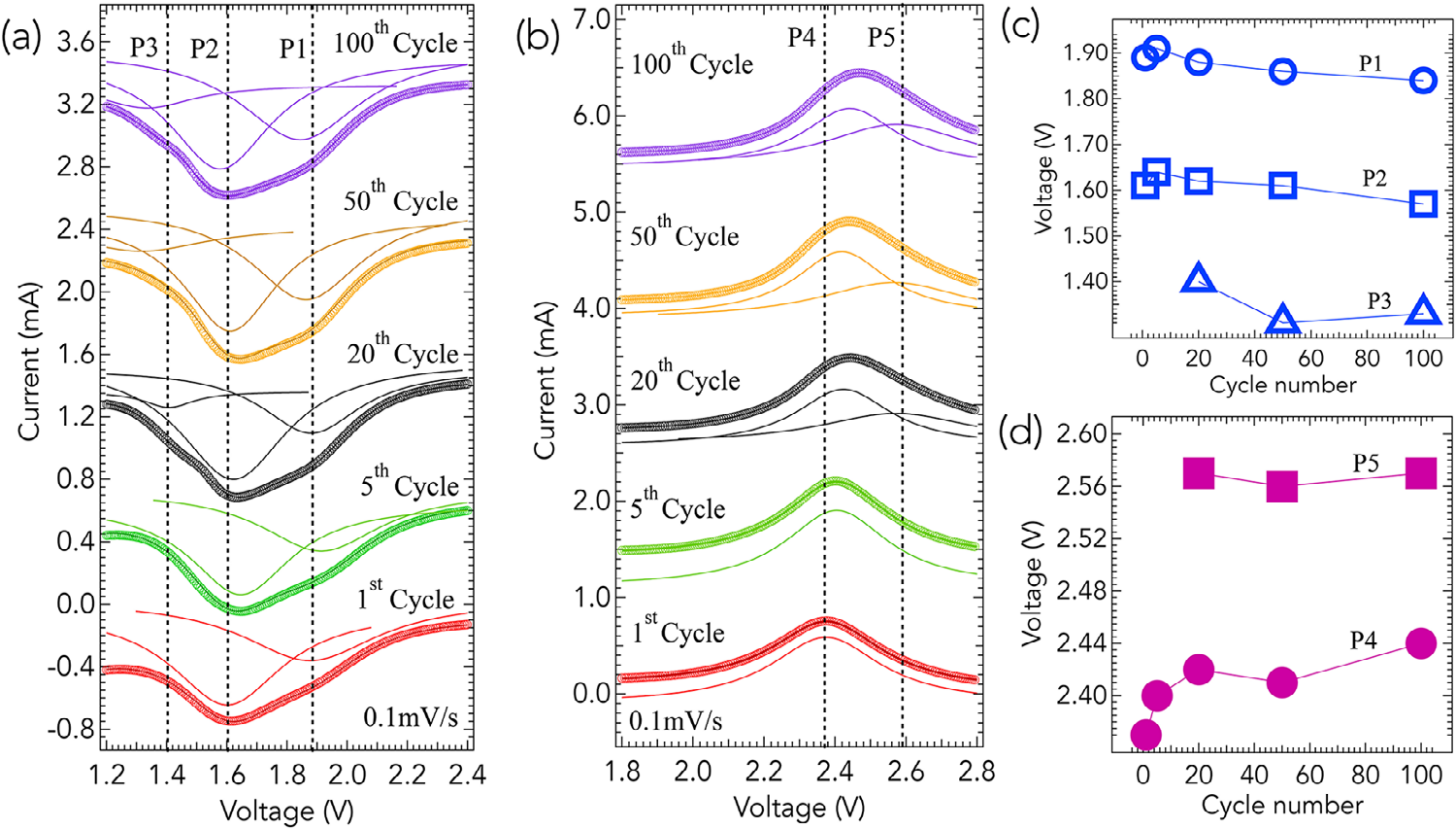
Cyclic voltammetry analysis of SPAN‐35 cathodes highlighting the evolution of redox peaks with cycling. a) Deconvoluted curves showing reduction features (P1–P3) at various cycles. The first reduction peak (P1) appears ≈1.9 V, followed by a prominent peak at 1.6 V (P2), and a shoulder at 1.4 V (P3) that emerges after the 20th cycle. These shifts suggest restructuring within the SPAN matrix and progressive formation of Li_2_S‐like domains. b) Oxidation features showing peaks at 2.4 V (P4) and a newly formed peak at 2.6 V (P5) from the 20th cycle. c) Evolution of the reduction peak voltages (P1–P3) as a function of cycle number, illustrating progressive downshifts consistent with structural transformation in S bonding. d) Evolution of oxidation peak voltages (P4 and P5) with cycling. P4 shows an increase in voltage up to 20 cycles followed by saturation, while P5 remains stable, indicating a rearranged redox‐active structure. These results corroborate the gradual transition from reversible S_*n*_ fragmentation to deeper lithiation and activation of the SPAN matrix.

SPAN‐35, containing ≈35 wt.% S, and commonly referred to as SPAN in the literature, exhibits redox characteristics that differ markedly from those of S_8_. **Figure** **2**a,b displays the deconvoluted CV curves of SPAN‐35 cathodes, with reduction and oxidation processes shown separately for detailed analysis. Notably, as seen in Figure 2a, SPAN lacks the cathodic peaks in the 2.1–2.4 V range observed in S_8_ (*cf*. Figure 1a), indicating that its reduction mechanism does not initiate from S_8_ nor proceed through the formation of long‐chain Li_2_S_*n*_ species (*cf*. Figure 1b).^[^ ^15^ ^]^ The deconvoluted CV curves reveal multiple overlapping peaks, suggesting a redox process that involves several intermediate stages. In the first cycle (bottom curve in Figure 2a), the initial major reduction peak appears at ≈1.9 V (labeled P1), followed by a more prominent peak at 1.6 V (P2). The 1.9 V peak likely corresponds to the cleavage of covalently bound S_*n*_ chains in C–S_*n*_–C motifs, where *n* > 2, while the 1.6 V peak is possibly associated with the formation of Li_2_SO_4_, as supported by our XPS analysis (discussed later in **Figure** **6**). Additionally, a shoulder peak emerges at ≈1.4 V (P3) after the 20th cycle. During oxidation, a single peak is observed at 2.4 V (P4) in the first cycle (bottom curve in Figure 2b). With continued cycling, a new oxidation peak emerges at 2.6 V (P5) after the 20th cycle, mirroring the new peak observed in reduction at 1.4 V.

**Figure 3:**
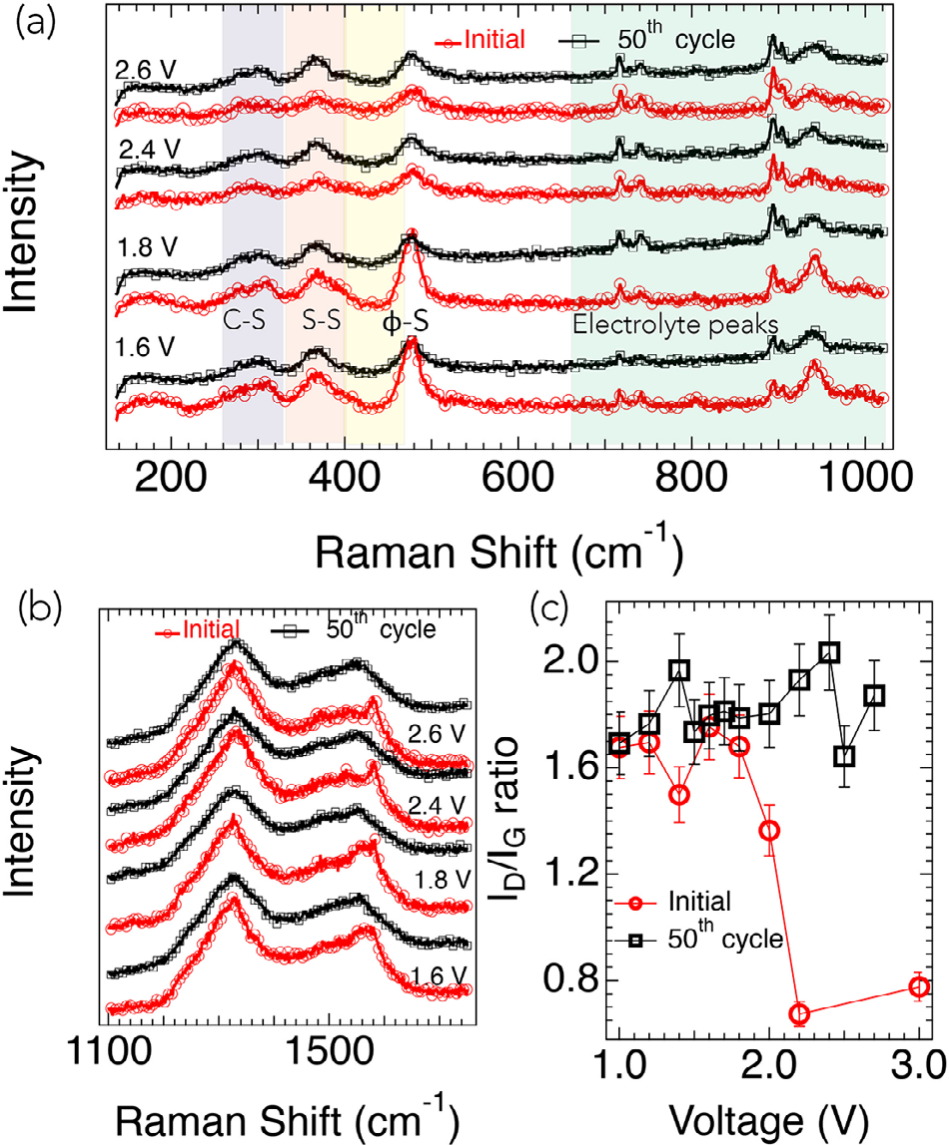
In situ Raman spectroscopy of SPAN‐35 cathodes during electrochemical cycling. a) Low‐frequency Raman spectra (200–1000 cm^−1^) at selected voltages during the initial (red circles) and 50th (black squares) cycles. Peaks at ≈305 cm^−1^ (shaded purple) and 375 cm^−1^ (shaded pink) correspond to C–S and S–S bending modes, respectively, while the 475 cm^−1^ peak (shaded yellow) corresponds to φ–S_*x*_ deformation (where φ denotes a carbon ring). The 475 cm^−1^ peak exhibits a pronounced intensity difference between reduction (1.6–1.8 V) and oxidation (2.4–2.6 V) in the initial cycle, indicating lithiation/delithiation of φ–S_*x*_ species. After 50 cycles, this intensity difference vanishes, suggesting reduced φ–S_*x*_ content and structural rearrangement. Peaks in the 650–950 cm^−1^ region (shaded green) arise from electrolyte species. b) High‐frequency Raman spectra (1100–1800 cm^−1^) highlighting the *D*‐band (1200–1400 cm^−1^) and *G*‐band (1450–1650 cm^−1^) features associated with the carbon backbone. c) Voltage‐dependent evolution of the *I*_*D*_/*I*_*G*_ ratio during the initial and 50th cycles. Sharp changes in *I*_*D*_/*I*_*G*_ in the initial cycle reflect structural rearrangement in the SPAN matrix, whereas the ratio remains stable after 50 cycles, confirming stabilization of the carbon backbone.

**Figure 4:**
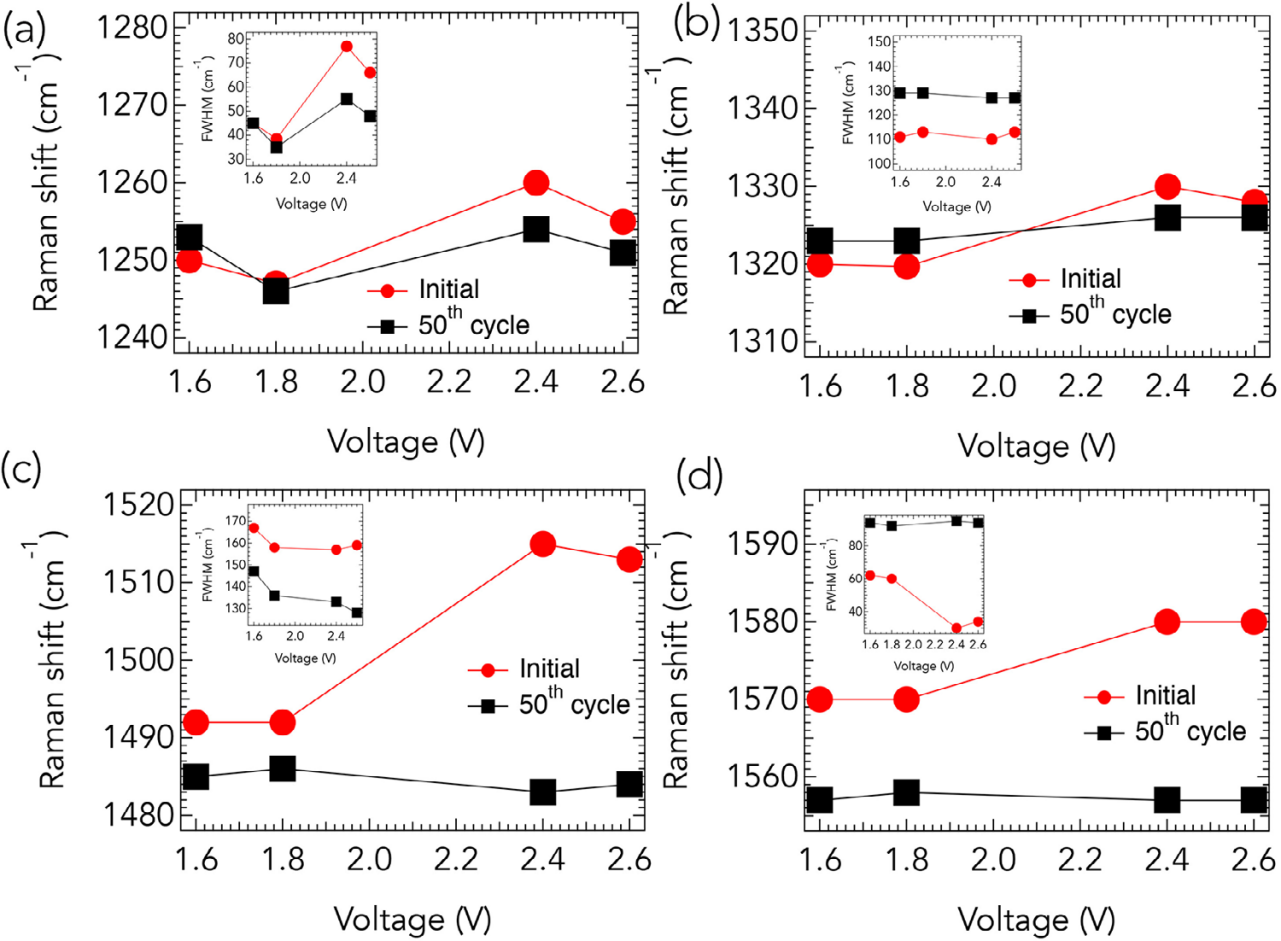
Raman peak position and full‐width at half maximum (FWHM) evolution for SPAN‐35 cathodes at different voltages during the initial and 50th cycles. a,b) Voltage‐dependent Raman shift and FWHM (inset) for the two components of the D‐band (1200–1400 cm^−1^). Minimal shifts are observed in the D‐band with cycling, indicating stable disorder‐related vibrational modes. c,d) Voltage‐dependent Raman shift and FWHM (inset) for the two components of the G‐band (1450–1650 cm^−1^). In the initial cycle, both G‐band peaks exhibit significant shifts and narrowing of FWHM with increasing voltage, consistent with charge‐transfer and strain‐induced effects in the *sp* ^2^ carbon backbone. After 50 cycles, both G‐band components stabilize at lower wavenumbers ( 1490 and 1560 cm^−1^) with minimal voltage dependence, indicating the formation of nanocrystalline *sp* ^2^ domains and structural stabilization of the carbon network.

**Figure 5:**
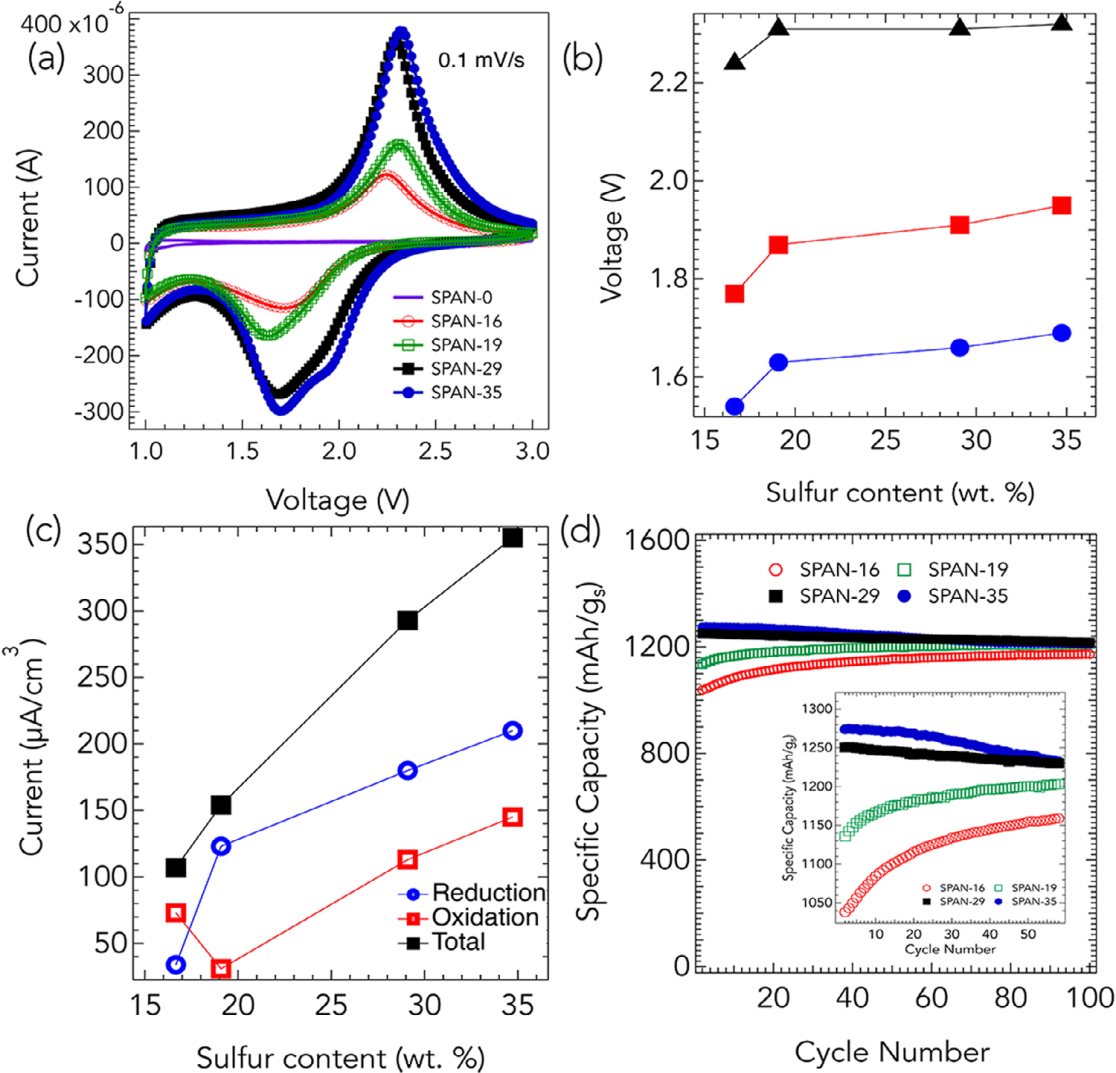
Electrochemical behavior of SPAN samples with varying sulfur content. a) CV curves of SPAN‐0, SPAN‐16, SPAN‐19, SPAN‐29, and SPAN‐35 at 0.1 mV s^−1^. SPAN‐0 exhibits no discernible redox features, while increasing sulfur content leads to the emergence of sharper, well‐defined peaks, particularly in SPAN‐29 and SPAN‐35. b) Voltage corresponding to peak reduction (circles), oxidation (squares), and overall redox window (triangles) as a function of sulfur content. Higher sulfur content correlates with higher peak voltages, consistent with the formation of longer S_*x*_ chains. c) Extracted reduction, oxidation, and total current density as a function of sulfur content, showing a near‐linear increase in redox activity with sulfur loading. d) Specific capacity vs. cycle number for all SPAN samples cycled at 0.5 C rate. SPAN‐16 and SPAN‐19 initially show lower specific capacities but gradually increase to match SPAN‐29 and SPAN‐35 after 100 cycles. This evolution is attributed to structural rearrangements that increase sulfur accessibility in low‐sulfur‐content samples. Inset: Zoom‐in of specific capacity data from 0 to 50 cycles.

**Figure 6:**
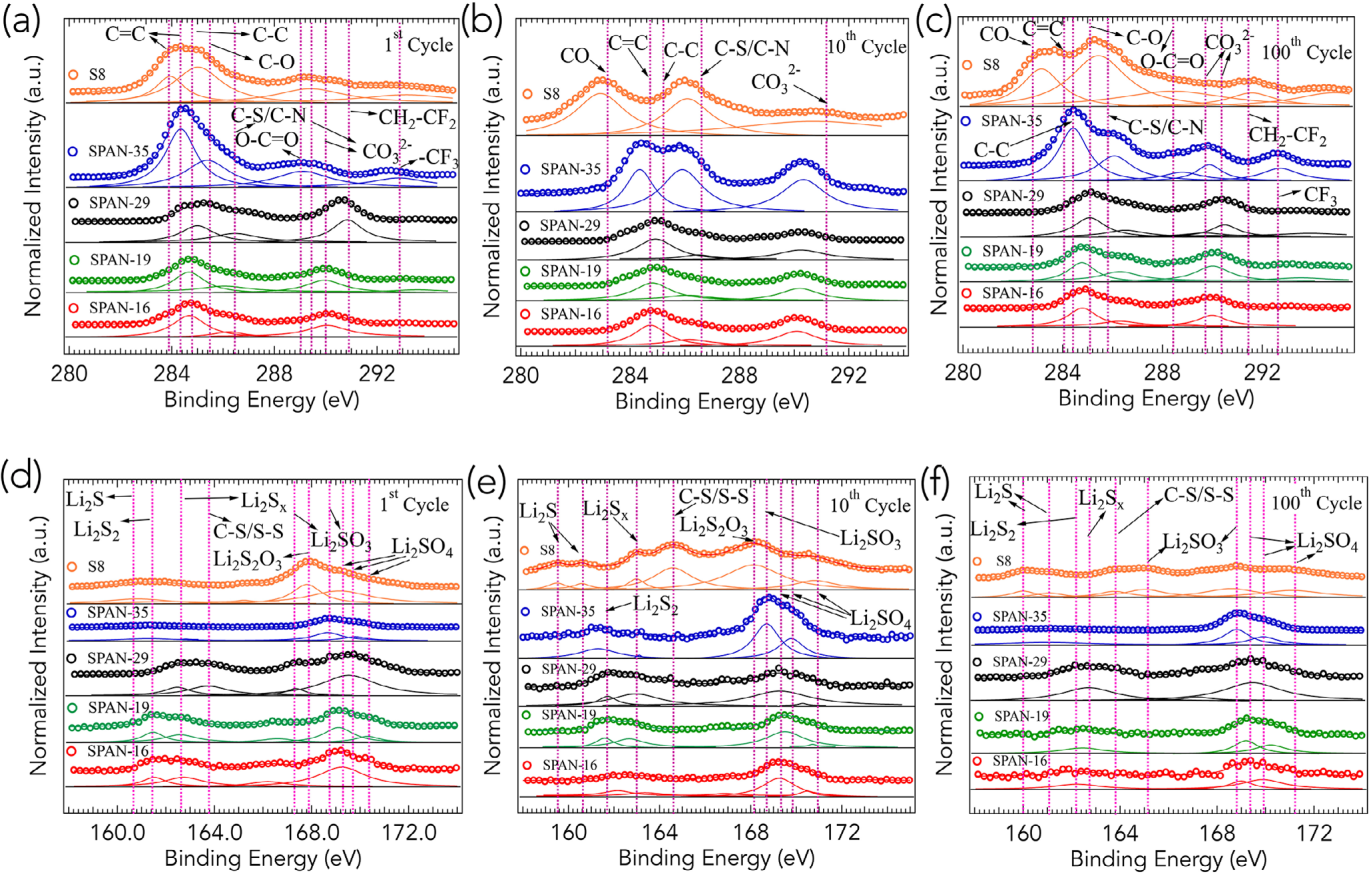
X‐ray photoelectron spectroscopy (XPS) analysis of SPAN and S_8_ cathodes over electrochemical cycling. a–c) C 1s spectra of S_8_, SPAN‐16, SPAN‐19, SPAN‐29, and SPAN‐35 after the (a) 1st, (b) 10th, and (c) 100th cycles. In SPAN samples, peaks at 284–285 eV correspond to C = C and C–C bonds, while peaks at 286–286.5 eV indicate C–S/C–N interactions, and 289.5–290.5 eV indicate CO_3_^2–^. Higher C 1s binding energies in SPAN‐16 and SPAN‐19 suggest increased *sp* ^3^ carbon content at low sulfur loading. Over cycling, the C–S/C–N peak intensifies, indicating increased S–C interactions, while CO_3_^2–^ remains prominent. d–f) S 2p spectra for the same samples and cycles. In the first cycle (d), SPAN samples exhibit peaks corresponding to Li_2_S_2_, Li_2_S_*x*_, and Li_2_SO_4_. SPAN‐29 shows an additional C–S/S–S peak at 163.8 eV. Upon cycling, the Li_2_S_2_ peak shifts and oxidized species (Li_2_SO_3_, Li_2_SO_4_) dominate after 100 cycles (f), indicating incorporation of sulfone groups in SPAN. S_8_ shows significant degradation and formation of surface decomposition products, unlike SPAN, where sulfur remains more stably bound. All samples were thoroughly washed, and measurements were repeated to confirm intrinsic chemical evolution.

Over successive cycles, a slight but noticeable shift is observed in the voltages of both the reduction (P1–P3) and oxidation (P4 and P5) peaks, as shown in Figure 2c,d. These shifts indicate potential changes in the electrochemical environment or the nature of the active material. Specifically, the 1.9 V and 1.6 V reduction peaks (P1 and P2) exhibit a progressive downshift with continued cycling, suggesting a gradual restructuring of C and S within the SPAN matrix. The new reduction peak, which emerges at 1.4 V (P3) beginning from the 20th cycle, also shifts to lower voltages upon further cycling (Figure 2c). This behavior is consistent with the formation and growth of Li_2_S‐like domains, which typically display cathodic peaks in the 1.2–1.4 V range. The appearance and evolution of P3 signal a gradual transition from reversible S_*n*_ fragmentation to deeper lithiation associated with Li_2_S formation. Concomitantly, the first oxidation peak (P4) exhibits an increase in voltage over the first 20 cycles, after which it saturates (Figure 2d). Additionally, a new oxidation peak at 2.6 V (P5) emerges from the 20th cycle, though this peak remains relatively stable in voltage with continued cycling.

We attribute the observed shifts in Figure 2c,d to structural changes in the SPAN matrix induced by cycling, which can be corroborated by in situ Raman spectroscopy. A comparison of in situ Raman spectra of SPAN‐35 cathodes after the initial cycle and the 50th cycle is presented in **Figure** **3**. This comparison is motivated by Figures 2c and d, where the net voltage shifts of all peaks (P1–P5) are most pronounced within the first 50 cycles. Consequently, we infer that the structural evolution of the SPAN cathode largely saturates by the 50th cycle.

The in situ Raman spectra recorded during electrochemical cycling exhibit several low‐frequency bands in the 300–500 cm^−1^ range (Figure 3a), providing insight into the S bonding environments within the cathode. Specifically, peaks near 305 and 375 cm^−1^ are observed, corresponding to the symmetric and asymmetric bending modes of C–S and S–S bonds, respectively. As discussed in our previous study,^[^ ^15^ ^]^ the peak at 475 cm^−1^ is associated with φ‐S_*x*_ deformation, where φ denotes a carbon ring. No significant change is observed in the 305 and 375 cm^−1^ peaks after 50 cycles. However, the 475 cm^−1^ peak shows a notable change in intensity. During the initial cycle, a strong Raman signal at 475 cm^−1^ is observed at 1.6 and 1.8 V, corresponding to the lithiation of φ‐S_*x*_ species. Furthermore, a marked difference in the intensity of this peak is evident between reduction (1.6 and 1.8 V) and oxidation (2.4 and 2.6 V) during the initial cycle. This suggests that the pristine SPAN matrix contains a substantial fraction of φ‐S_*x*_ species, which undergo lithiation in the 1.6–1.8 V range and delithiation in the 2.4–2.6 V range. After 50 cycles, no discernible difference is observed in the intensity of the 475 cm^−1^ peak between reduction (1.6 and 1.8 V) and oxidation (2.4 and 2.6 V), indicating that the SPAN matrix has undergone rearrangement, resulting in a reduced population of φ‐S_*x*_ species. This spectral evolution strongly correlates with the emergence of new peaks at 1.4 V (P3) and 2.6 V (P5) in Figure 2. The decline of φ‐S_*x*_ states upon cycling facilitates the formation of Li_2_S, which is typically observed in the 1.2–1.4 V range. It should be noted that the Raman bands in the 650–950 cm^−1^ region in Figure 3a arise from the electrolyte.

While the low‐frequency Raman peaks provide insights into S bonding environments, Raman features in the 1100–1800 cm^−1^ region are sensitive to structural rearrangements in the C backbone of SPAN (Figure 3b). This spectral region exhibits two broad features: the disorder (*D*) band between 1200–1400 cm^−1^ and the graphitic (*G*) band between 1450–1650 cm^−1^. Detailed fitting of the *D*‐ and *G*‐bands at various voltages during the initial and 50th cycles is shown in Figure S4 (Supporting Information). For all spectra, we employed a four‐peak fitting procedure with two peaks for the *D*‐band region and two for the *G*‐band region. The ratio of *D*‐band to *G*‐band intensity (*I*_*D*_/*I*_*G*_) is commonly used as a metric to quantify the degree of disorder in carbon materials.^[^ ^33^ ^]^ This ratio was computed from the fitted spectra (Figure S4, Supporting Information) by dividing the total integrated area of the two *D*‐band peaks (1200–1400 cm^−1^) by that of the two *G*‐band peaks (1450–1650 cm^−1^). In our previous study of in situ Raman spectra of SPAN‐35,^[^ ^15^ ^]^ we observed sharp variations in *I*_*D*_/*I*_*G*_ during the initial charge–discharge cycle, attributed to significant structural rearrangements in the SPAN matrix (Figure 3c). However, such pronounced changes in *I*_*D*_/*I*_*G*_ were absent during the 50th cycle, confirming that substantial modifications in the C backbone occur primarily during the early stages of cycling.

The *D*‐ and *G*‐band peak positions along with their full‐width at half maximum (FWHM) for the initial and 50th cycles are presented in **Figure** **4**. The total height of the *y*‐axis is fixed at 40 cm^−1^ for all panels in Figure 4, facilitating direct comparison of the magnitude of peak shifts across different bands and cycles. In the initial cycle, the two peaks within the *D*‐band region (Figure 4a,b) exhibit minimal shifts in their peak positions, whereas the peaks within the *G*‐band region (Figure 4c,d) display more pronounced shifts. Importantly, the trends in *D*‐band peak positions and FWHM remain largely unchanged after 50 cycles. In contrast, the two peaks in the *G*‐band region show substantial variations in both peak position and FWHM as a function of voltage during the initial cycle (Figure 4c,d). Specifically, a sharp peak at ≈1580 cm^−1^ is observed at 2.6 V (Figure 4d; Figure S4, Supporting Information), corresponding to the *sp* ^2^ graphitic network within the C backbone of SPAN. The FWHM of this peak decreases from 60 cm^−1^ at 1.6 and 1.8 V to 30 cm^−1^ at 2.4 and 2.6 V in the initial cycle (see inset, Figure 4d). These changes are consistent with charge‐transfer and strain‐induced Raman shifts associated with lithiation and delithiation in *sp* ^2^ C. Following 50 cycles, both *G*‐band components are downshifted to ≈1490 cm^−1^ and 1560 cm^−1^, which is indicative of the formation of nanocrystalline *sp* ^2^ domains within the SPAN matrix. Moreover, in the 50th cycle, the peak positions of both *G*‐band components remain relatively stable with respect to voltage, further supporting structural stabilization upon cycling. These observations imply that the C backbone in SPAN actively participates in lithiation and delithiation, which is in agreement with theoretical predictions.^[^ ^25^, ^26^ ^]^

Overall, the observed trends in *I*_*D*_/*I*_*G*_, together with the voltage‐ and cycle‐dependent evolution of the *D*‐ and *G*‐bands, corroborate the electrochemical evidence for gradual restructuring of the C backbone in SPAN. This restructuring is marked by an increase in nanocrystalline *sp* ^2^ domains and a concurrent reduction in φ‐S_x_ content. These findings are further supported by voltage‐ and cycle‐resolved electrochemical impedance spectroscopy (EIS), shown in Figures S5 and S6 (Supporting Information). All EIS data were fitted using the equivalent circuit model presented in Figure S7 (Supporting Information), and the corresponding fitting parameters are summarized in Table S1 (Supporting Information). Analysis of the EIS data at various voltages and cycles reveals that the series resistance *R*_*s*_ of the electrode remains essentially unchanged over 200 cycles (Figure S8a, Supporting Information), ruling out electrode resistance as the origin of the voltage shifts observed for P1–P5 in Figure 2. In contrast, the charge transfer resistance *R*_*CT*_ decreases significantly from values exceeding 200 Ω in the initial cycle to 20–60 Ω after 50 cycles (Figure S8b, Supporting Information). This trend suggests that the redox‐active sites involved in Li binding undergo chemical restructuring and activation during the early stages of cycling, as evidenced by CV, Raman spectroscopy, and EIS.

To further investigate the role of S bonding within SPAN, we synthesized samples with varying S content. The C and S composition of all SPAN samples was determined via CHSNO analysis, as summarized in Table 1. A key observation from the CHSNO data is the progressive decrease in C content as S content increases from 16 to 35 wt.%. Specifically, SPAN‐16 and SPAN‐19 exhibit the highest C concentrations at 54.5 and 55.07 wt.%, respectively, while SPAN‐29 and SPAN‐35 contain lower C fractions at 48.5 and 43.3 wt.%. This trend suggests that at higher S concentrations, S progressively replaces carbon‐rich domains in the backbone through direct bonding. Based on previous studies, we hypothesize that shorter –S_*x*_– chains with *x* ⩽ 3 are prevalent at sulfur contents below 20 wt.%, while longer chains with *x* ⩽ 7 emerge at higher sulfur concentrations.^[^ ^15^ ^]^

The CV curves for all SPAN samples are shown in **Figure** **5**a. SPAN‐0, which contains no sulfur, exhibits no discernible peaks or valleys, consistent with its electrochemical inactivity. Upon S incorporation, SPAN‐16 and SPAN‐19 begin to display broad reduction features, indicative of short C–S_*n*_–C chains and modest redox activity. In the initial cycle, notable differences in the CV curve shape within the 1.5–2.0 V window are observed between SPAN‐16/19 and SPAN‐29/35, indicating distinct C–S bonding environments. In SPAN‐29 and SPAN‐35, the CV profiles exhibit two sharper and more well‐defined peaks with clear separation in the 1.5–2.0 V region. The voltages corresponding to both reduction and oxidation processes shift to higher potentials with increasing S content (Figure 5b). At lower S content, SPAN‐16 and SPAN‐19 predominantly contain C–S_*n*_–C bonds with *n* ⩽ 3, resulting in lower peak voltages since the reduction potential of Li_2_S_*n*_ decreases with decreasing *n*. The observed shift to higher peak voltages with increasing sulfur content suggests the formation of longer S chains in SPAN‐29 and 35. While the relative contributions of reduction and oxidation peaks vary with S content, the total capacity increases nearly linearly with S content (Figure 5c). Despite this, we expect the specific capacity normalized to S mass (per gram of S) to remain similar across all SPAN samples. Interestingly, as shown in Figure 5d, the initial specific capacity of SPAN‐16 and SPAN‐19 is significantly lower than that of SPAN‐29 and SPAN‐35. However, upon cycling at 0.5 C rate, SPAN‐16 and SPAN‐19 exhibit a gradual increase in specific capacity, ultimately reaching values comparable to SPAN‐29 and SPAN‐35 after 100 cycles. We attribute this increase to enhanced utilization of S in SPAN‐16 and SPAN‐19 over successive cycles. According to CHSNO data (Table 1), SPAN‐16 and SPAN‐19 have higher C content, which likely renders a portion of S initially inaccessible. Structural rearrangements in the C backbone during cycling (as discussed in Figures 2, 3, 4) enable increased accessibility of S, thereby leading to improved capacity. Similar trends were observed at various C‐rates (0.5–4 C), as shown in Figure S9 (Supporting Information).

We employed XPS to investigate the surface chemistry and the evolution of chemical states in all SPAN and S_8_ samples over various charge–discharge cycles (Figure 6). The XPS spectra of the as‐prepared powders are provided in Figure S10 (Supporting Information) of SI. Detailed peak assignments are summarized in **Table** **2** based on prior XPS studies.^[^ ^34^, ^35^, ^36^, ^37^, ^38^, ^39^, ^40^, ^41^, ^42^, ^43^, ^44^, ^45^, ^46^ ^]^ The C 1*s* spectra offer insights into the carbon environment within the SPAN samples (Figure 6a–c). In the initial cycle, all SPAN samples exhibit a primary peak ≈284–285 eV, accompanied by minor peaks near 286–286.5 eV (C–S/C–N) and 289.5–290.5 eV (CO_3_^2–^). SPAN‐35 displays a distinct peak at 284.3 eV, corresponding to C = C bonds in *sp* ^2^ C, along with a peak at 285.2 eV associated with *sp* ^3^ carbon.^[^ ^47^ ^]^ In contrast, SPAN‐16, 19, and 29 exhibit slightly upshifted C 1*s* peaks at 285.1 and 286.2 eV, suggesting a higher fraction of *sp* ^3^ C in samples with lower S content. By the 10^th^ cycle, all SPAN samples show an increase in the intensity of the C–S/C–N peak (286.1–286.4 eV), indicating enhanced interaction between C and S within the SPAN matrix. By the 100th cycle, the CO_3_^2–^ peak remains prominent in all samples, while minor shifts are observed in the C–S/C–N peak positions. The C 1*s* spectra of S_8_ cathodes differ significantly, as the carbon signal arises primarily from the conductive filler. These spectra are characterized by dominant peaks at 284.0 eV (C = C) and 289.5 eV (CO_3_^2–^). Upon cycling, additional peaks emerge ≈285.6 eV (C–O) and 288.6 eV (O–C = O), reflecting surface degradation and electrolyte decomposition.^[^ ^2^, ^14^, ^15^, ^16^, ^17^, ^18^, ^19^, ^20^ ^]^

**Table 2:** XPS peaks for SPAN and S_8_ samples.

Sample	Element	1^*st*^ Cycle	10^*th*^ Cycle	100^*th*^ Cycle
		(Position (eV),	(Position (eV),	(Position (eV),
		Assignment, ref)	Assignment, ref)	Assignment, ref)
SPAN‐16	S2p	161.40 (Li_2_S_2_)^[^ ^26^ ^]^	162.10 (Li_2_S_2_)^[^ ^25^ ^]^	162.10 (Li_2_S_2_)^[^ ^25^ ^]^
		162.70 (Li_2_S_*x*_)^[^ ^33^ ^]^	163.00 (Li_2_S_*x*_)^[^ ^33^ ^]^	169.10 (Li_2_SO_4_)^[^ ^36^ ^]^
		169.30 (Li_2_SO_4_)^[^ ^36^ ^]^	169.30 (Li_2_SO_4_)^[^ ^36^ ^]^	169.90 (Li_2_SO_4_)^[^ ^36^ ^]^
	C1s	284.80 (C‐C)^[^ ^34^ ^]^	284.80 (C‐C)^[^ ^34^ ^]^	284.80 (C‐C)^[^ ^34^ ^]^
		290.00 (${\mathrm{CO}}_{3}^{2-}$)^[^ ^33^ ^]^	286.10 (C‐S/C‐N)^[^ ^39^ ^]^	286.40 (C‐S/C‐N)^[^ ^37^ ^]^
			290.20 (${\mathrm{CO}}_{3}^{2-}$)^[^ ^33^ ^]^	289.80 (${\mathrm{CO}}_{3}^{2-}$)^[^ ^25^ ^]^
SPAN‐19	S2p	161.40 (Li_2_S_2_)^[^ ^26^ ^]^	161.60 (Li_2_S_2_)^[^ ^26^ ^]^	162.30 (Li_2_S_2_)^[^ ^34^ ^]^
		162.70 (Li_2_S_*x*_)^[^ ^33^ ^]^	162.70 (Li_2_S_*x*_)^[^ ^33^ ^]^	169.30 (Li_2_SO_4_)^[^ ^36^ ^]^
		169.30 (Li_2_SO_4_)^[^ ^36^ ^]^	169.30 (Li_2_SO_4_)^[^ ^36^ ^]^	170.20 (Li_2_SO_4_)^[^ ^34^ ^]^
	C1s	284.80 (C‐C)^[^ ^34^ ^]^	284.80 (C‐C)^[^ ^34^ ^]^	284.80 (C‐C)^[^ ^34^ ^]^
		290.00 (${\mathrm{CO}}_{3}^{2-}$)^[^ ^33^ ^]^	286.10 (C‐S/C‐N)^[^ ^39^ ^]^	286.40 (C‐S/C‐N)^[^ ^37^ ^]^
			290.20 (${\mathrm{CO}}_{3}^{2-}$)^[^ ^33^ ^]^	289.80 (${\mathrm{CO}}_{3}^{2-}$)^[^ ^25^ ^]^
SPAN‐29	S2p	162.40 (Li_2_S_2_)^[^ ^34^ ^]^	161.60 (Li_2_S_2_)^[^ ^26^ ^]^	162.80 (Li_2_S_*x*_)^[^ ^33^ ^]^
		163.80 (C‐S/S‐S)^[^ ^35^ ^]^	163.00 (Li_2_S_*x*_)^[^ ^33^ ^]^	169.50 (Li_2_SO_4_)^[^ ^36^ ^]^
		167.20 (Li_2_SO_3_)^[^ ^32^ ^]^	169.30 (Li_2_SO_4_)^[^ ^36^ ^]^	
		169.70 (Li_2_SO_4_)^[^ ^36^ ^]^		
	C1s	285.00 (C‐C)^[^ ^38^ ^]^	284.80 (C‐C)^[^ ^34^ ^]^	285.00 (C‐C)^[^ ^38^ ^]^
		286.40 (C‐S/C‐N)^[^ ^37^ ^]^	286.40 (C‐S/C‐N)^[^ ^37^ ^]^	286.40 (C‐S/C‐N)^[^ ^37^ ^]^
		290.90 (CH_2_‐CF_2_)^[^ ^35^ ^]^	290.20 (${\mathrm{CO}}_{3}^{2-}$)^[^ ^33^ ^]^	290.50 (${\mathrm{CO}}_{3}^{2-}$)^[^ ^34^ ^]^
SPAN‐35	S2p	161.40 (Li_2_S_2_)^[^ ^26^ ^]^	161.20 (Li_2_S_2_)^[^ ^26^ ^]^	161.00 (Li_2_S_2_)^[^ ^26^ ^]^
		168.70 (Li_2_SO_3_)^[^ ^36^ ^]^	168.70 (Li_2_SO_3_)^[^ ^36^ ^]^	168.80 (Li_2_SO_3_)^[^ ^36^ ^]^
		169.70 (Li_2_SO_4_)^[^ ^36^ ^]^	169.80 (Li_2_SO_4_)^[^ ^36^ ^]^	169.90 (Li_2_SO_4_)^[^ ^36^ ^]^
	C1s	284.40 (C = C)^[^ ^38^ ^]^	284.40 (C = C)^[^ ^38^ ^]^	284.40 (C = C)^[^ ^38^ ^]^
		285.50 (C‐O)^[^ ^33^ ^]^	286.00 (C‐S/C‐N)^[^ ^39^ ^]^	286.20 (C‐S/C‐N)^[^ ^39^ ^]^
		288.90 (O‐C = O)^[^ ^34^ ^]^	290.40 (${\mathrm{CO}}_{3}^{2-}$)^[^ ^34^ ^]^	288.60 (O‐C = O)^[^ ^38^ ^]^
		292.90 (‐CF_3_)^[^ ^34^ ^]^		289.80 (${\mathrm{CO}}_{3}^{2-}$)^[^ ^25^ ^]^
				292.50 (‐CF_3_)^[^ ^33^ ^]^
S8	S2p	160.70 (Li_2_S)^[^ ^25^ ^]^	159.60 (Li_2_S)^[^ ^26^ ^]^	160.20 (Li_2_S)^[^ ^32^ ^]^
		167.90 (Li_2_S_2_O_3_)^[^ ^37^ ^]^	160.60 (Li_2_S)^[^ ^33^ ^]^	161.00 (Li_2_S_2_)^[^ ^26^ ^]^
		169.30 (Li_2_SO_4_)^[^ ^36^ ^]^	163.00 (Li_2_S_*x*_)^[^ ^33^ ^]^	163.80 (C‐S/S‐S)^[^ ^35^ ^]^
			164.60 (C‐S/S‐S)^[^ ^26^ ^]^	165.20 (Li_2_S_2_O_4_)^[^ ^36^ ^]^
			168.20 (Li_2_S_2_O_3_)^[^ ^37^ ^]^	168.30 (Li_2_S_2_O_3_)^[^ ^37^ ^]^
			170.80 (Li_2_SO_4_) ^[^ ^34^ ^]^	171.20 (Li_2_SO_4_) ^[^ ^34^ ^]^
	C1s	283.70 (CO)^[^ ^42^ ^]^	283.00 (CO)^[^ ^40^ ^]^	283.20 (CO)^[^ ^41^ ^]^
		285.00 (C‐C)^[^ ^38^ ^]^	286.10 (C‐S/C‐N)^[^ ^39^ ^]^	285.40 (C‐O)^[^ ^33^ ^]^
		289.50 (${\mathrm{CO}}_{3}^{2-}$)^[^ ^25^ ^]^	290.40 (${\mathrm{CO}}_{3}^{2-}$)^[^ ^34^ ^]^	288.60 (O‐C = O)^[^ ^38^ ^]^
		293.00 (‐CF_3_)^[^ ^34^ ^]^		291.60 (CH_2_‐CF_2_)^[^ ^35^ ^]^

As shown in Figure 6d–f, S_8_ cathodes exhibit distinct S 2*p* peaks corresponding to long‐chain LiPS and sulfate species. In the first cycle, dominant peaks are observed at 160.7 eV (Li_2_S) and 167.9 eV (Li_2_S_2_O_3_). With continued cycling, significant degradation of the S_8_ cathodes is evident, as new peaks emerge at 164.6 eV (S–S) and 168.3 eV (Li_2_SO_3_). Notably, the peak intensity in the higher binding energy region (168–170 eV), corresponding to Li_2_SO_3/4_, decreases with cycle number in S_8_, in contrast to SPAN where such oxidized species remain relatively stable. The progressive decrease in oxidized S species in S_8_ suggests a dissolution–shuttle mechanism, which is largely absent in SPAN due to the retention of S within the polymer matrix. Our electrolyte formulation contains EC and DOL, both of which are susceptible to reductive decomposition during electrochemical cycling.^[^ ^48^ ^]^ As reported by Ota et al.^[^ ^49^ ^]^ and Aurbach et al.,^[^ ^50^ ^]^ EC degradation results in the formation of lithium alkyl carbonates (ROCO_2_Li) and Li_2_CO_3_, whereas DOL can undergo ring‐opening polymerization to yield polyethylene oxide (PEO)‐like species. These decomposition products are commonly identified as components of the solid electrolyte interphase (SEI). Furthermore, the reduction of LiTFSI in ether‐based solvents has been shown to produce S–O compounds such as Li_2_S_2_O_4_ and Li_2_SO_3_ in the outer SEI, along with Li_2_S in the inner layers, as confirmed by XPS.^[^ ^49^ ^]^ Importantly, these oxygenated species can form despite the absence of O in the TFSI^−^ anion itself, highlighting that solvent decomposition and interfacial reactions at the cathode surface are the primary sources of O incorporation into the SEI.

All SPAN samples exhibit characteristic S 2*p* peaks corresponding to Li_2_S_2_, intermediate sulfur species (Li_2_S_*x*_), and oxidized sulfur species (Li_2_SO_3/4_) (Figures 6d‐f). The exact binding energy positions vary slightly across different SPAN compositions. Specifically, SPAN‐16, SPAN‐19, and SPAN‐29 show S 2*p* peaks at ≈161.4 eV (Li_2_S_2_), 162.7 eV (Li_2_S_*x*_), and 168.5–170.5 eV (Li_2_SO_3/4_), indicating the coexistence of reduced and oxidized S species. Additionally, SPAN‐29 exhibits a peak at 163.8 eV (C–S/S–S) during the first cycle. Upon extended cycling, notable shifts in the S 2*p* peaks are observed. By the 10th cycle, the Li_2_S_2_ peak in SPAN‐16 shifts to 162.1 eV, while in SPAN‐19 it shifts to 161.6 eV, suggesting gradual evolution in S bonding environments. Similar trends are observed in SPAN‐29 and SPAN‐35, where the Li_2_S_2_ peaks stabilize ≈161.6 eV and 161.2 eV, respectively. By the 100^th^ cycle, all SPAN samples are dominated by oxidized sulfur species, with Li_2_SO_3/4_ peaks appearing prominently in the 168.5–170.5 eV range.^[^ ^40^ ^]^ To confirm that the observed oxidized S species are intrinsic to the SPAN structure and not derived from the electrolyte, all samples were thoroughly washed with DOL, and XPS measurements were repeated on at least three independent cells for each cycle. The relative intensity of Li_2_SO_3/4_ in the higher binding energy region (168–170 eV) consistently exceeds that of Li_2_S/Li_2_S_2_ in the lower binding energy region (160–164 eV) across all SPAN samples. In contrast, S_8_ cathodes do not exhibit such behavior upon extended cycling. These observations suggest that the SPAN structure incorporates S–O groups, which become increasingly dominant with cycling. A detailed correlation between CV, Raman, and XPS peak positions is provided in Table S2 (Supporting Information).

**Figure 7:**
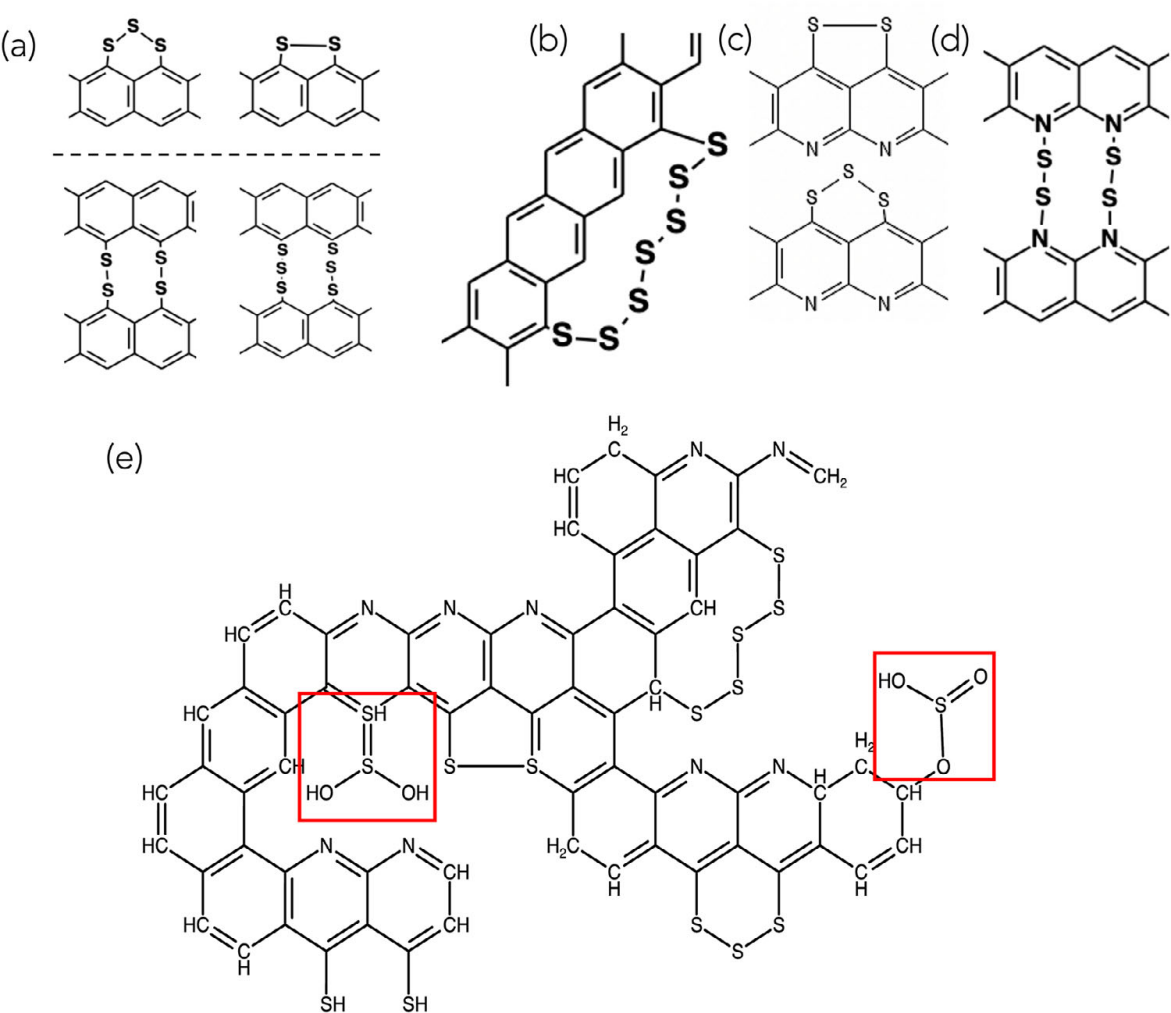
a) Previously proposed structural configurations for SPAN with short sulfur chains (S_2_, S_3_) linked to single aromatic rings or bridging adjacent rings. b) Long sulfur chains (S_*x*_, *x* > 3) extending across multiple aromatic rings, which become prevalent at higher sulfur content SPAN. c) Proposed SPAN structures involving nitrogen atoms in the polymer backbone forming N–S bonds. d) Polypyridine‐like network incorporating N–S bonding motifs in SPAN. e) Newly proposed SPAN structure based on comprehensive CV, Raman, and XPS analysis, incorporating short sulfur chains (S_2_, S_3_) and sulfone functionalities (highlighted in red boxes), which were not considered in previous models. This structure was validated for valence accuracy using RDKit, a cheminformatics toolkit for molecular modeling.^[^ ^51^ ^]^

Previously, various molecular structures for SPAN (**Figures** **7**a‐d) have been proposed based on a range of characterization techniques, including electrochemical signatures, NMR, XPS, IR, and Raman spectroscopy.^[^ ^2^, ^13^, ^14^, ^15^, ^16^, ^17^, ^18^, ^19^, ^20^, ^27^ ^]^ At low S content, short S chains (S_2_, S_3_) are typically linked to a single hexagonal carbon ring or serve as a bridge between adjacent rings (Figure 7a). As the sulfur content increases, longer sulfur chains (S_*x*_, where *x* > 3) are more likely to form, extending across multiple rings, as depicted in Figure 7b. Additionally, the incorporation of N atoms in SPAN has been proposed to lead to the formation of alternative networks (Figure 7c), including polypyridine‐like structures containing N–S bonds (Figure 7d). Based on a comprehensive analysis of CV, Raman, and XPS data discussed in Figures 1‐6, we propose a revised molecular structure for SPAN, shown in Figure 7e, which includes short S chains (S_2_, S_3_) in conjunction with S–O functionalities that were not considered in previous models. This proposed structure was validated for valence consistency using RDKit, a widely used cheminformatics toolkit for molecular modeling and structural validation.^[^ ^51^ ^]^

Our results suggest that SPAN structure evolves from initial discharge with the growth of nanocrystalline *sp* ^2^ domains and changes in S bonding, as discussed above. Given the strong involvement of the C backbone seen in Raman studies (*cf*. Figures 3 and 4), we expect that the contribution of surface‐controlled (or capacitive) processes to the total capacity matches or dominates the diffusion‐controlled (or redox) processes in SPAN. We performed scan rate‐dependent CV based on the Trasatti method to obtain crucial insights into charge storage mechanisms of SPAN electrodes. By analyzing current response as a function of the scan rate (*v*), it is possible to differentiate between diffusion and surface‐controlled charge storage processes. The Trasatti method relies on the Cottrell equation,^[^ ^15^ ^]^ which describes the diffusion‐limited response.

(1) $$i=nFACD^{1/2}v^{-1/2}$$

where *i* is the current, *n* is the number of electrons, *F* is the Faraday constant, *A* is the electrode surface area, *C* is the concentration, *D* is the diffusion coefficient, and *v* is the scan rate. While the diffusion‐controlled contribution changes as $v^{\frac{1}{2}}$, the capacitive contributions scale linearly with *v*. The decomposition of total charge into diffusion‐limited and capacitive contributions reveals whether bulk diffusion processes dominate charge storage or if a significant fraction of charge is stored at the electrode surface. We note that this method assumes a clear separation between surface‐limited and diffusion‐limited contributions, but in redox‐active systems like SPAN, mechanisms such as pseudocapacitive redox, confined diffusion within polymeric domains, and charge transfer at localized heteroatoms may partially overlap. The method also assumes relatively uniform ion access to electrochemically active sites. However, variations in pore size distribution, local tortuosity, and binder‐induced occlusion can modulate ion transport in ways that are not purely “diffusion‐limited” in the classical sense. In this context, the observed capacitive contribution could be underestimated if surface sites are not fully accessible within the experimental scan rate window.

**Figure 8:**
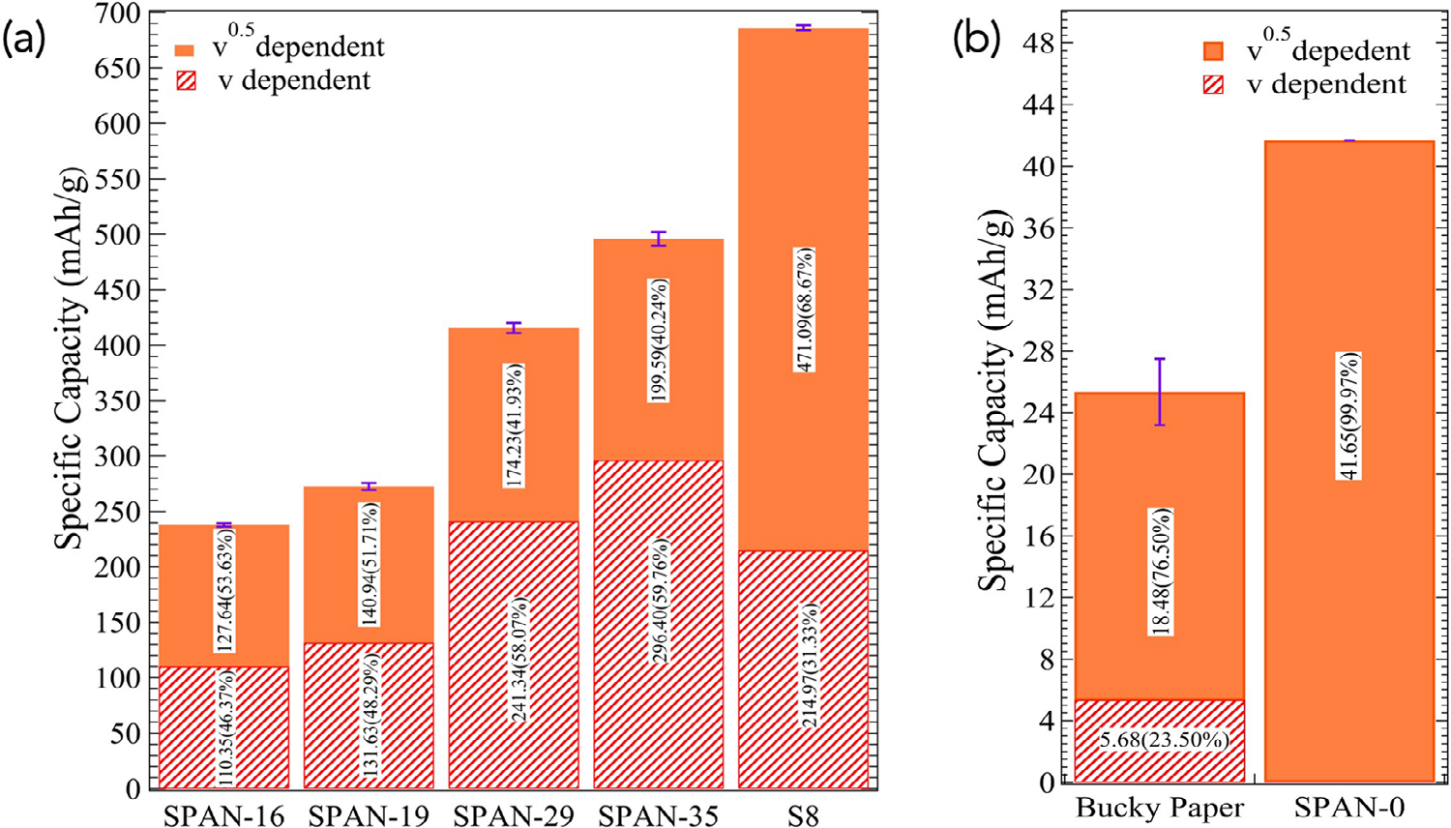
Trasatti's method was applied to quantify the surface and diffusion contributions to the specific capacity, which is normalized by the total material weight. a) Capacity bar plot of SPAN‐16, 19, 29, 35 and S_8_ and b) Bucky‐paper and SPAN‐0 electrodes.

**Figure** **8** shows the specific capacity contributions divided into *v*‐dependent (surface‐limited) and *v* ^1/2^‐dependent (diffusion‐controlled) processes for SPAN electrodes, S_8_, SPAN‐0, and Bucky Paper (made using entangled multi‐walled carbon nanotubes or CNTs) for comparison. For SPAN‐35, the *v* ^1/2^‐dependent contribution was 200 mAh g^−1^, accounting for 40% of the total specific capacity of 495 mAh g^−1^ (normalized by total material weight). This component represents the diffusion‐controlled transport of Li ions from the electrolyte to the electrode surface and subsequently into the bulk of the SPAN matrix. The remaining 60%, represented by the *v*‐dependent contribution, reflects surface‐limited and intercalation‐like lithium storage processes similar to graphitic carbon. For example, as shown in Figure 8b, CNTs exhibited 25 % *v*‐dependent capacity due to Li intercalation while SPAN‐0 did not show such a feature. As evidenced by our spectroscopic studies (*cf*. Figures 3 and 4), the C backbone in SPAN facilitates Li intercalation. Like SPAN‐35, SPAN‐29 also showed 41 % *v* ^1/2^‐dependent contribution, and a dominant *v*‐dependent contribution, suggesting that surface or intercalation‐like processes are the primary mechanisms. SPAN‐16 and 19 exhibited a higher a *v* ^1/2^‐dependent contribution of 50% of total capacity. Such increased diffusion‐controlled storage relative to SPAN‐29 and 35 suggests that the C backbone differs significantly. This was confirmed in the XPS data (*cf*. Figure 6) where SPAN‐35 showed the presence of C = C bonds in *sp* ^2^ C 284.3 eV while other SPAN samples showed slightly higher peaks 285–285.3 eV related to C‐C bonds in *sp* ^3^ C. Unlike SPAN, S_8_ showed a *v* ^1/2^‐dependent contribution of 471 mAh g^−1^, comprising 69% of its total specific capacity of 700 mAh g^−1^. This high proportion highlights the bulk diffusion‐controlled behavior typical of elemental sulfur, where Li ions react with S to form LiPS (*cf*. Figure 1b). However, the absence of a significant *v*‐dependent contribution indicates that S_8_ lacks the surface interactions or intercalation‐like behavior characteristic of SPAN. The observed differences between the surface *vs*. diffusion‐controlled process between SPAN and S_8_ samples can be explained, using the redox density of states, as discussed below.

In electrochemistry, changes in the chemical state through electron exchange are described by the redox reaction $\text{ox}+e^{-}⇌\text{red}$. The free energy of the reaction is given by $\Delta G_{r}=e\left(V-V_{r}\right)=\bar{\mu }-E_{r}$, where *V* is the electrode potential, *V*_*r*_ is the formal potential of the redox system, $\bar{\mu }$ is the electrochemical potential of the electrons in the electrode or current collector, and *E*_*r*_ is the electrochemical potential of electrons in the redox system. It should be noted that the electrochemical potential ($\bar{\mu }$) is related to the chemical potential (μ) as $\bar{\mu }=\mu +ze\phi$, where ϕ is the inner potential and *z* is the oxidation/reduction number. For unperturbed or neutral systems (ϕ = 0), the chemical and electrochemical potentials are equal in the zero‐temperature limit.

Let us consider the redox system at a stationary external potential outside the redox window (*V*_*o*_). This corresponds to the “fully occupied” ground state electron density in the neutral state $\rho ({\bar{\mu }}_{o})$, where electrochemical potential ${\bar{\mu }}_{o}=eV_{o}$. Upon the application of a small sinusoidal perturbation at *V*_*o*_ (e.g., as in EIS), the spatial electronic density can be perturbed without the exchange of electrons or redox reactions $\text{i.e.,}\int_{\Omega }\delta \rho \left(\vec{r}\right)d\vec{r}=0$. In DFT, the ground state electron density ρ(*r*) of an *N*‐electron system in a region Ω with ∫_Ω_ρ(*r*)*d* **r** = *N*, under the influence of a fixed external potential *v*(*r*), minimizes the energy functional

(2) $$\begin{array}{ccc} E_{v}\left[\rho \right] & = & T_{s}\left[\rho \right]+\int_{\Omega }\rho \left(r\right)v\left(r\right)dr+\frac{1}{2}\int \int_{\Omega \times \Omega }\frac{e^{2}\rho \left(r\right)\rho \left(r^{\prime }\right)}{4\pi \varepsilon_{0}\left|r-r^{\prime }\right|}drdr^{\prime }\\ & & +\;E_{xc}\left[\rho \right] \end{array}$$

where *T*_*s*_[ρ] is the kinetic energy of the noninteracting electron system and *E*_*xc*_[ρ] is the exchange‐correlation energy.

The variational condition δ*E*_*v*_[ρ] = 0 leads to

(3) $$\begin{array}{c} \int_{\Omega }\delta \rho \left(r\right)\left\{\frac{\delta T_{s}\left[\rho \right]}{\delta \rho (r)}+v\left(r\right)+\int_{\Omega }\frac{e^{2}\rho \left(r^{\prime }\right)}{4\pi \varepsilon_{0}\left|r-r^{\prime }\right|}dr^{\prime }+\frac{\delta E_{xc}\left[\rho \right]}{\delta \rho (r)}\right\}dr=0 \end{array}$$

where δρ(*r*) is an arbitrary variation of ρ(*r*) satisfying ∫_Ω_δρ(*r*)*d* **r** = 0. The above equation requires that the expression in the braces be a constant independent of ρ. Now, consider a voltage inside the redox window (*V*_*i*_) where the redox reactions occur leading to a change in the number of electrons to *N*′ and the electron density to ρ′(*r*).

For the correct density of the *N*′‐electron system with ∫_Ω_ρ′(*r*)*d* **r** = *N*′, the expression in the braces of Equation (3) is also a constant. A subtraction gives

(4) $$\begin{array}{ccc} \left\{\frac{\delta T_{s}\left[\rho^{\prime }\right]}{\delta \rho^{\prime }\left(r\right)}-\frac{\delta T_{s}\left[\rho \right]}{\delta \rho (r)}\right\} & + & \int_{\Omega }\frac{e^{2}\left[\rho^{\prime }\left(r^{\prime }\right)-\rho \left(r^{\prime }\right)\right]}{4\pi \varepsilon_{0}\left|r-r^{\prime }\right|}dr^{\prime }\\ & + & \left\{\frac{\delta E_{xc}\left[\rho^{\prime }\right]}{\delta \rho^{\prime }\left(r\right)}-\frac{\delta E_{xc}\left[\rho \right]}{\delta \rho (r)}\right\}=\text{const} \end{array}$$

The variation in kinetic energy and the exchange‐correlation energy of the electron gas with density changes is expected to be small for large redox active molecules on an ideal conductor.^[^ ^52^, ^53^, ^54^ ^]^ In such a case, the only non‐zero term ∫_Ω_[*e*Δρ(*r*′)/4πε_0_|**r** − **r**′|]*d* **r**′ can be equated to a redox capacitor with a potential of *Q*/*C*_*r*_, where *C*_*r*_ is the redox capacity resulting from exchange of electrons and *Q* = (*N*′ − *N*)*e* is the exchanged charge. Hence by analogy, we may write the constant in Equation (3) as (*N*′ − *N*)*e* ^2^/*C*_*r*_, and for any **r** ∈ Ω, we have

(5) $$\int_{\Omega }\frac{e^{2}\rho^{\prime }\left(r^{\prime }\right)}{4\pi \varepsilon_{0}\left|r-r^{\prime }\right|}dr^{\prime }-\int_{\Omega }\frac{e^{2}\rho \left(r^{\prime }\right)}{4\pi \varepsilon_{0}\left|r-r^{\prime }\right|}dr^{\prime }=\frac{\left(N^{\prime }-N\right)e^{2}}{C}$$

The constant *C* = *C*(*N*, *N*′) is defined as the redox capacitance of the system. A detailed derivation can be found in refs. [54, 55]. Assuming that the variations in the density are not sharp during the charging or discharging process, we write

(6) $$E\left[\rho^{\prime }\right]-E\left[\rho \right]=\sum_{N+1}^{N^{\prime }}\varepsilon_{i}^{KS}+\frac{{\left(N^{\prime }-N\right)}^{2}e^{2}}{C_{r}}$$

where *E*[ρ] is the energy functional at a charge density ρ and $\varepsilon_{i}^{KS}$ are the energies of the Kohn–Sham states. The change in the internal chemical potential of the redox system with respect to the metallic current collectors itself (i.e., with respect to an infinite density of electronic states whose small change is negligible during the charging/discharging) can be quantified by $\Delta \mu =\frac{N^{\prime }-N}{g_{r}\left(\mu \right)}$, where *g*_*r*_(μ) is the electrochemical density of states of the redox system. By comparing with Equation (5), we see that *C*_*r*_ = *e* ^2^ *g*_*r*_(μ). The first term in Equation (6) is related to the electrostatic capacitance as $\frac{\gamma }{C_{q}}=\sum_{N+1}^{N^{\prime }}\varepsilon_{i}^{KS}$, where γ is a constant depending on *N*′ − *N* and *C*_*q*_ is the electronic quantum capacitance.

The expression *C*_*r*_ = *e* ^2^ *g*_*r*_(μ) highlights that the redox capacitance *C*_*r*_ is governed by the redox density of states *g*_*r*_(μ), analogous to how electronic quantum capacitance in low‐dimensional conductors is governed by the electronic density of states *D*(*E*_*F*_). In systems such as graphene, for example, the quantum capacitance is given by *C*_*q*_ = *e* ^2^ *D*(*E*_*F*_), where a low *D*(*E*_*F*_) near the Dirac point leads to poor charge accommodation and voltage instability. Similarly, a low *g*_*r*_(μ) implies that even a small change in charge (*N*′ − *N*) causes a large shift in chemical potential Δμ, which may manifest as sharp voltage transitions during charge/discharge.

In contrast, a broad and continuous *g*_*r*_(μ), spread over the electrochemical window of interest, allows for smoother potential changes and enhanced cycling stability. This is particularly important for redox‐active systems such as SPAN, where the sulfur species exist in a variety of covalently bound chain lengths (S_*x*_, 1 ⩽ *x* ⩽ 7), resulting in a distributed ensemble of redox states. We hypothesize that this structural heterogeneity gives rise to a broader redox DOS compared to elemental S_8_, which typically is expected to exhibit discrete redox steps associated with polysulfide intermediates. A broadened *g*_*r*_(μ) in SPAN would therefore facilitate gradual charge accommodation and improved voltage stability akin to how metallic systems with a wide *D*(*E*_*F*_) support larger quantum capacitance.

**Figure 9:**
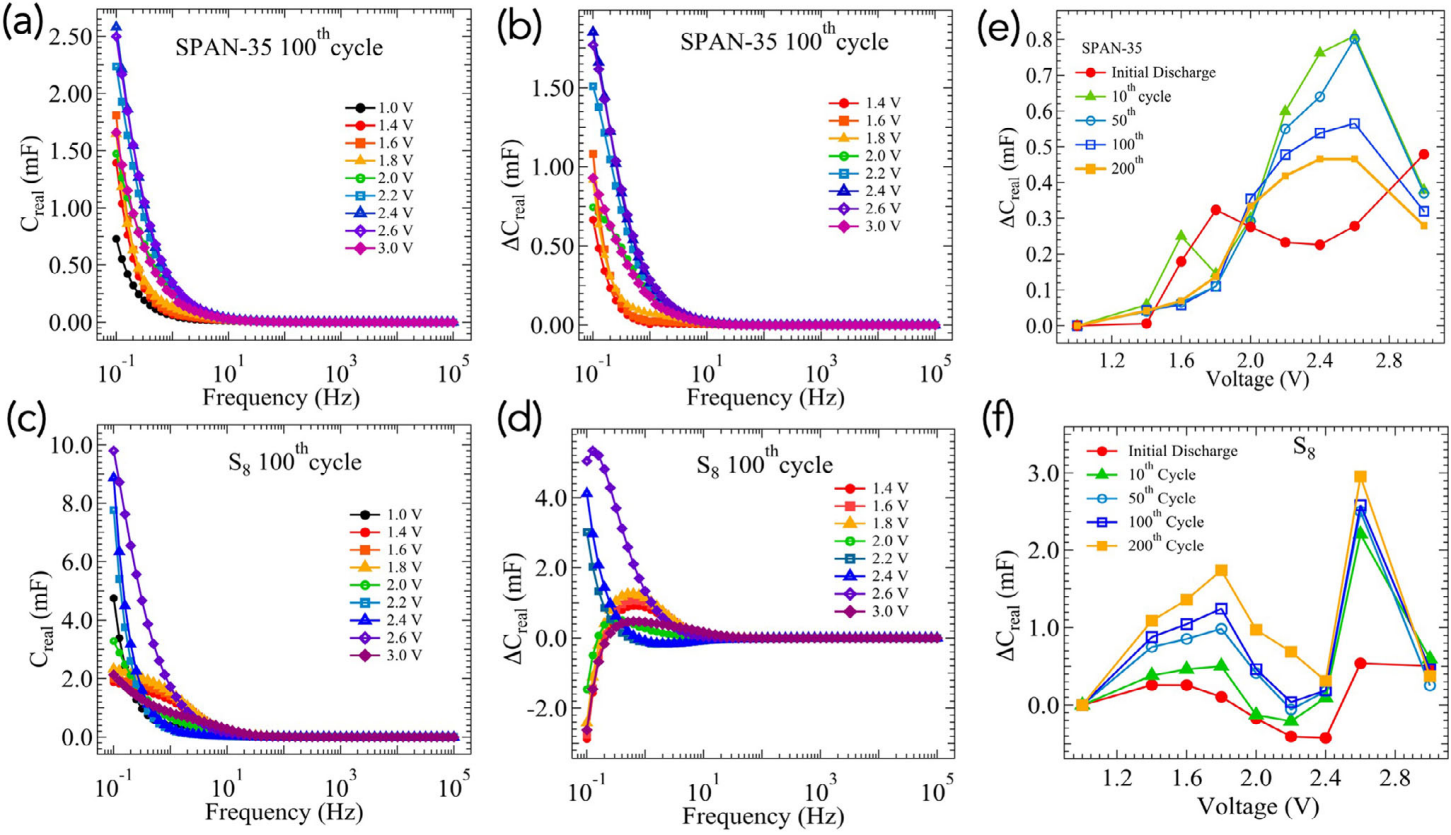
The redox density of states (*g*_*r*_(μ)) was measured using electrochemical capacitance spectroscopy. a,b) show C_real_ and Δ*C*_real_ as a function of frequency for SPAN‐35 electrodes, while c,d) display C_real_ and Δ*C*_real_ as a function of frequency for S_8_ electrode. Panels e,f) show Δ*C*_real_ as a function of voltage for SPAN‐35 and S_8_ electrodes, respectively.

Electrochemical capacitance spectroscopy (ECS) enables an experimental determination of *g*_*r*_(μ) by quantifying the differential capacitance as a function of applied potential.^[^ ^52^, ^53^, ^55^ ^]^ The measured capacitance consists of two key components discussed in Equations (5) and (6): the redox and electrostatic capacitance. The redox component is directly related to *g*_*r*_(μ), which can be extracted experimentally by measuring capacitance *C*(*V*) and isolating only the redox contributions through careful subtraction of the non‐Faradaic contributions by placing the electrode outside the redox window. The complex Z*(ω) (impedance) function is converted into complex C*(ω) (capacitance) by $C^{*}\left(\omega \right)=\frac{1}{j\omega Z^{*}\left(\omega \right)}$, where ω is the angular frequency and $j=\sqrt{-1}$ (complex number). As discussed in refs. [52, 53, 55], Z*(ω) sampled across a range of frequencies at a potential is phasorially converted into complex capacitance C*(ω) with real (*C*_*real*_ = φ*Z*″) and imaginary (*C*_*imag*_ = φ*Z*′), where φ = (ω|*Z*|^2^)^−1^ and |*Z*| is the modulus of Z*. To extract *g*_*r*_(μ), *C*_*real*_(ω) is measured as a function of frequency (**Figure** **9**) with the electrode placed at a voltage both inside (*V*_*i*_) and outside (*V*_*o*_) of the redox window, as determined by CV discussed in Figures 2 and 5. A subtraction at a low frequency ω_0_ gives Δ*C*_*real*_(Figure 9), which is directly proportional to *g*_*r*_(μ).

Figure 9a–f presents *C*_real_ and Δ*C*_real_ for SPAN‐35 and S_8_ at different voltages and frequencies. In SPAN‐35, the evolution of *C*_real_ over multiple cycles exhibits a voltage‐dependent distribution that shifts slightly as cycling progresses, indicating structural and electronic modifications within the C‐S framework. Such changes in the C‐S bonds were also observed in the XPS and Raman (*cf*. Figures 3, 4, and 6). The *g*_*r*_(μ)remains relatively stable beyond the 50th cycle, highlighting the robustness of SPAN's redox‐active network. For S_8_, Δ*C*_real_ (Figure 9f) shows a distinct frequency dependence, with pronounced variations at specific voltages. Unlike SPAN, *g*_*r*_(μ) in S_8_ is highly dynamic in the polysulfide voltage window 1.2–2.0 V and changes considerably across cycles (Figure 9f), reflecting the formation and dissolution of polysulfide species during charge‐discharge cycles. The stable redox behavior in SPAN underscores its pseudocapacitive nature, wherein S redox reactions occur in a confined manner without dissolution into the electrolyte. We also measured Δ*C*_real_ for all SPAN samples (Figure S11, Supporting Information), which shows the evolution of *g*_*r*_(μ) as a function of S content. Our results suggest that the *g*_*r*_(μ) varies very slightly across SPAN samples despite their difference in C and S content. Such an observation is consistent with the stable charge‐discharge observed in all SPAN samples (*cf*. Figure 5d). In summary, as expected, all SPAN samples exhibited a broader redox density of states *g*_*r*_(μ) compared to S_8_, consistent with the presence of diverse S chain motifs (S_*x*_, 1 ⩽ *x* ⩽ 7) and S–O motifs. While S_8_ displays a sharp and localized redox peak centered ≈2.6 V, the redox DOS for SPAN spans a wider voltage range from 2.0 to 2.8 V, reflecting a continuum of redox transitions enabled by the heterogeneous bonding environment.

## 4 Conclusion

In this study, we systematically investigated the charge storage mechanisms in sulfurized polyacrylonitrile (SPAN) cathodes with sulfur contents ranging from 0 to 35 wt.%. Our results demonstrate that SPAN exhibits fundamentally different electrochemical behavior compared to elemental sulfur (S_8_), enabled by its chemically bound sulfur species and active carbon backbone. Unlike S_8_, which displays diffusion‐limited redox behavior and pronounced polysulfide formation, SPAN cathodes show a dominant pseudocapacitive character with minimal polysulfide dissolution.

Cyclic voltammetry (CV) revealed that SPAN‐35 exhibits sharp and well‐defined redox peaks centered ≈1.6–2.6 V, with surface‐controlled pseudocapacitive contributions constituting ≈60% of the total capacity, as quantified by Trasatti analysis. This surface contribution was higher in SPAN‐29/35 (≈60%) and decreased to 50% in SPAN‐16 and SPAN‐19, indicating that increased sulfur content promotes pseudocapacitive behavior through more accessible redox‐active sites.

In situ Raman spectroscopy showed that SPAN undergoes significant structural evolution within the first 50 cycles, marked by the decline of ϕ–S_*x*_ species (identified via the 475 cm^−1^ peak) and the formation of nanocrystalline *sp* ^2^ carbon domains. These transformations were corroborated by voltage‐dependent shifts in the *D*‐ and *G*‐band Raman modes. X‐ray photoelectron spectroscopy (XPS) further revealed the emergence and persistence of S–O (Li_2_SO_3_/Li_2_SO_4_) functionalities across all SPAN samples, with S 2*p* peaks in the 168–170 eV range dominating after 100 cycles. These S–O species were stable and reproducible across multiple cells, suggesting intrinsic incorporation into the SPAN structure. Importantly, these oxygenated sulfur species, not previously reported in SPAN models, likely contribute to the broadening of redox activity and pseudocapacitive behavior.

Electrochemical capacitance spectroscopy (ECS) measurements provided direct quantification of the redox density of states, *g*_*r*_(μ), using a density functional theory (DFT)‐inspired framework. SPAN‐35 displayed a broad and stable *g*_*r*_(μ) across 2.0–2.8 V, in contrast to S_8_, which exhibited narrow, cycle‐dependent redox peaks centered near 2.6 V. The stability and breadth of *g*_*r*_(μ) in SPAN correlate with its structural heterogeneity—namely, diverse S_*x*_ chain motifs and S–O functionalities—and underlie its superior cycling stability and rate performance. Together, these findings establish a clear structure–function relationship in SPAN, wherein higher sulfur content, increased sp^2^ carbon domains, and incorporation of S–O groups contribute to a broad, stable redox density of states and enhanced pseudocapacitive charge storage.

## Conflict of Interest

The authors declare no conflict of interest.

## Supporting information

Supporting Information

## Data Availability

The data that support the findings of this study are available from the corresponding author upon reasonable request.
