# Supplementary material for: Broad Redox Density of States and S–O Functionalities Drive Stable Pseudocapacitive Behavior in Sulfurized Polyacrylonitrile (SPAN) Cathodes
Source: Adv Sci (Weinh). 2025 Sep 29;12(43):e11459. doi: 10.1002/advs.202511459 (PMC12631823; doi:10.1002/advs.202511459)
Supplement: Supplementary file 1 — Supporting Information [file ADVS-12-e11459-s001.pdf]

Supporting Information

Broad Redox Density of States and S–O Functionalities Drive Stable Pseudocapacitive Behavior in Sulfurized Polyacrylonitrile (SPAN) Cathodes

Sajib Kumar Mohonta, Nawraj Sapkota, and Ramakrishna Podila*

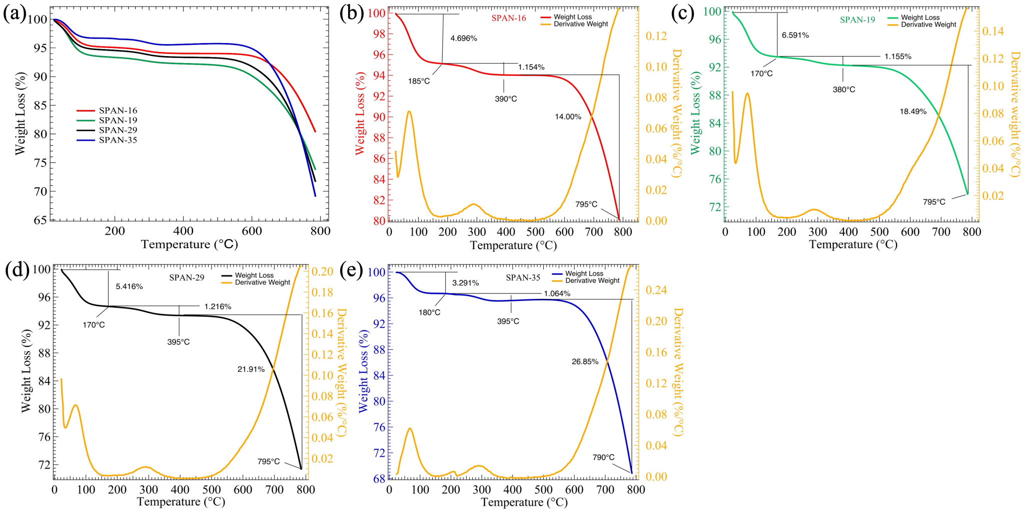

Figure S1: Thermogravimetric analysis (TGA) and derivative thermogravimetric (DTG) profiles of SPAN samples with different sulfur content: SPAN-16, SPAN-19, SPAN-29, and SPAN-35. (a) TGA curves showing overall weight loss with increasing temperature up to 800°C. Individual TGA and DTG for (b) SPAN-16, (c) SPAN-19, (d) SPAN-29, and (e) SPAN-35.

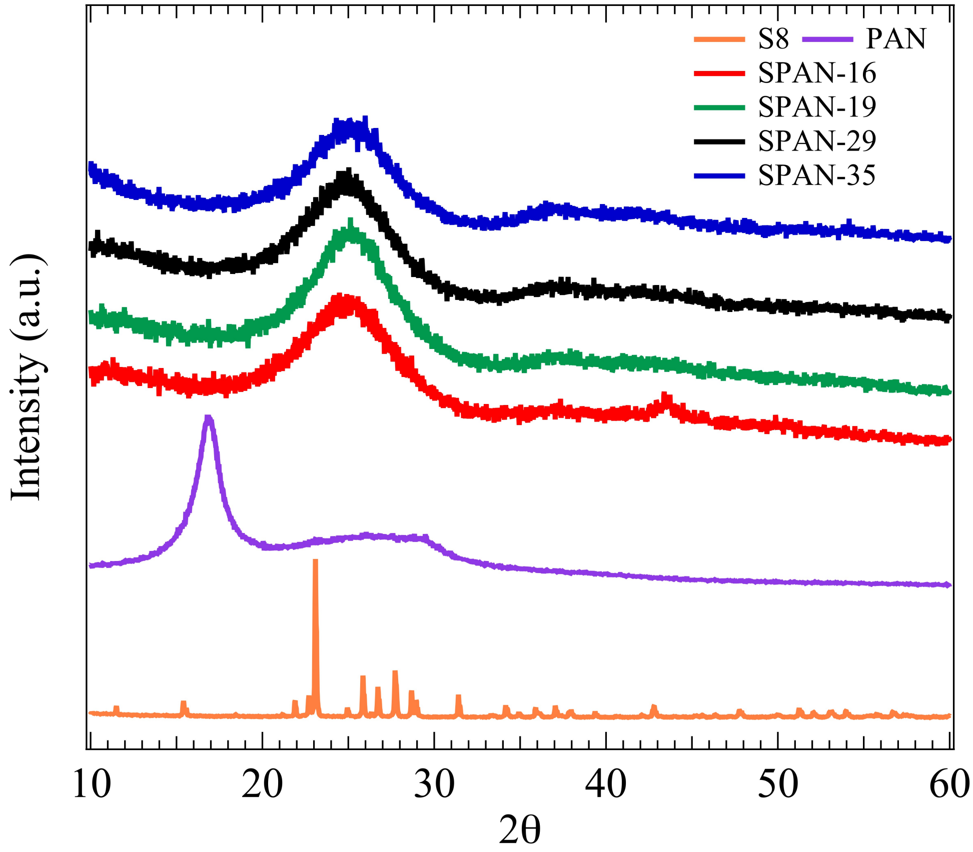

Figure S2: X-ray diffraction (XRD) patterns of elemental sulfur (S_8), polyacrylonitrile (PAN), and SPAN samples (SPAN-16, SPAN-19, SPAN-29, SPAN-35). The sharp peaks of S_8 confirm its high crystallinity. PAN exhibits a broad peak at 17° showing amorphous structure. For all SPAN samples, a broad peak $24 - 26°$ is observed. This can be attributed to the (002) plane of disordered carbon with short-range stacking of nanographitic domains or aromatic clusters.

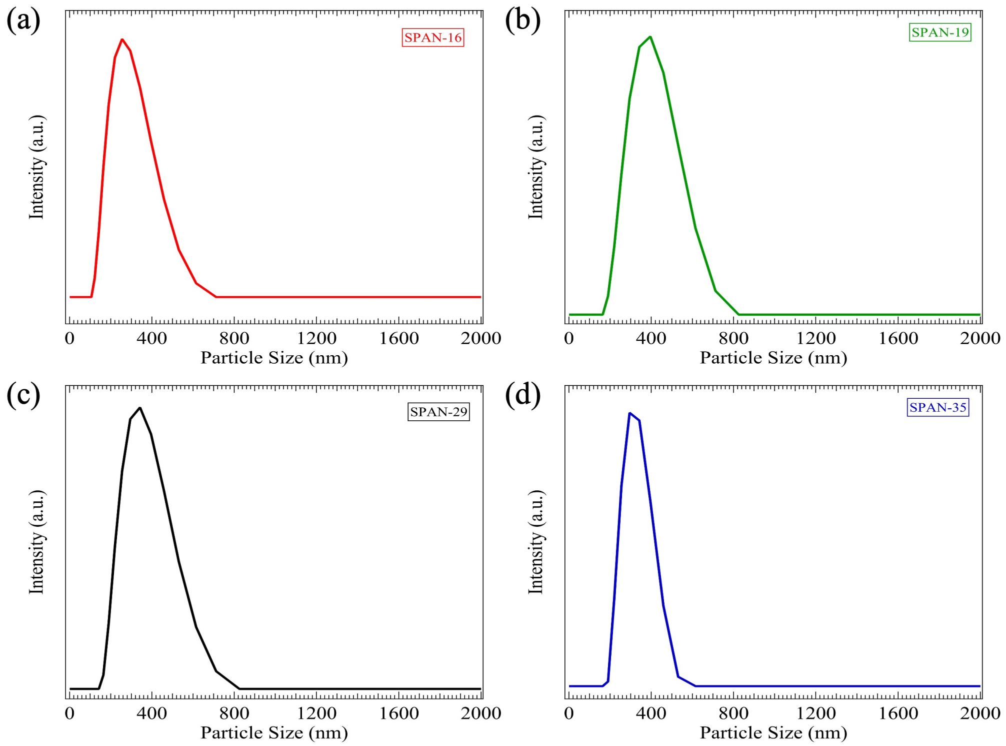

Figure S3: Dynamic Light Scattering (DLS) of SPAN samples suspended in isopropanol with varying sulfur content: (a) SPAN-16, (b) SPAN-19, (c) SPAN-29, and (d) SPAN-35.

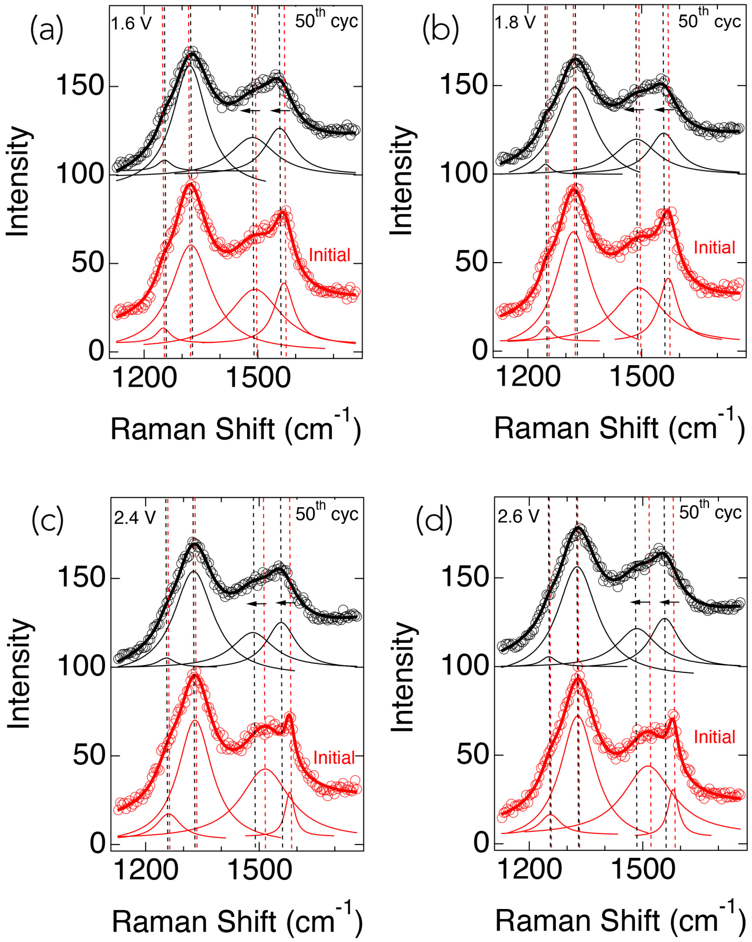

Figure S4: Detailed fitting of D- and G-bands at various voltages during the initial and 50th cycles at (a) 1.6 V, (b) 1.8 V, (c) 2.4 V, and (d) 2.6 V. For all spectra, we employed a four-peak fitting procedure with two peaks for the D-band region and two for the G-band region.

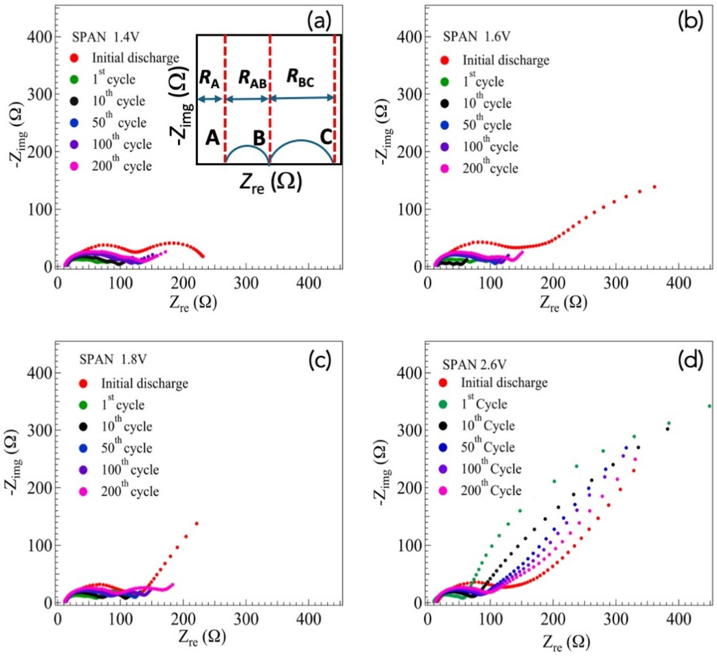

Figure S5: Nyquist plots of SPAN-35 electrodes measured at different potentials and cycle numbers: (a) 1.4 V, (b) 1.6 V, (c) 1.8 V, and (d) 2.6 V. Each panel displays impedance spectra recorded after the initial discharge (orange), and at the 1st (red), 10th (blue), 50th (green), 100th (black), and 200th (magenta) cycles. The inset in panel (a) illustrates the extraction of resistance values based on [1], where R_A is the ohmic resistance, R_{AB} the interfacial resistance, and R_{BC} the charge transfer and diffusion resistance. Panels (a)-(c) exhibit significant evolution in the shape and magnitude of the impedance features, indicative of potential- and cycle-dependent restructuring of interfacial redox sites. In contrast, panel (d) at 2.6 V shows minimal spectral evolution across cycles, suggesting stabilization of redox processes at higher potentials.

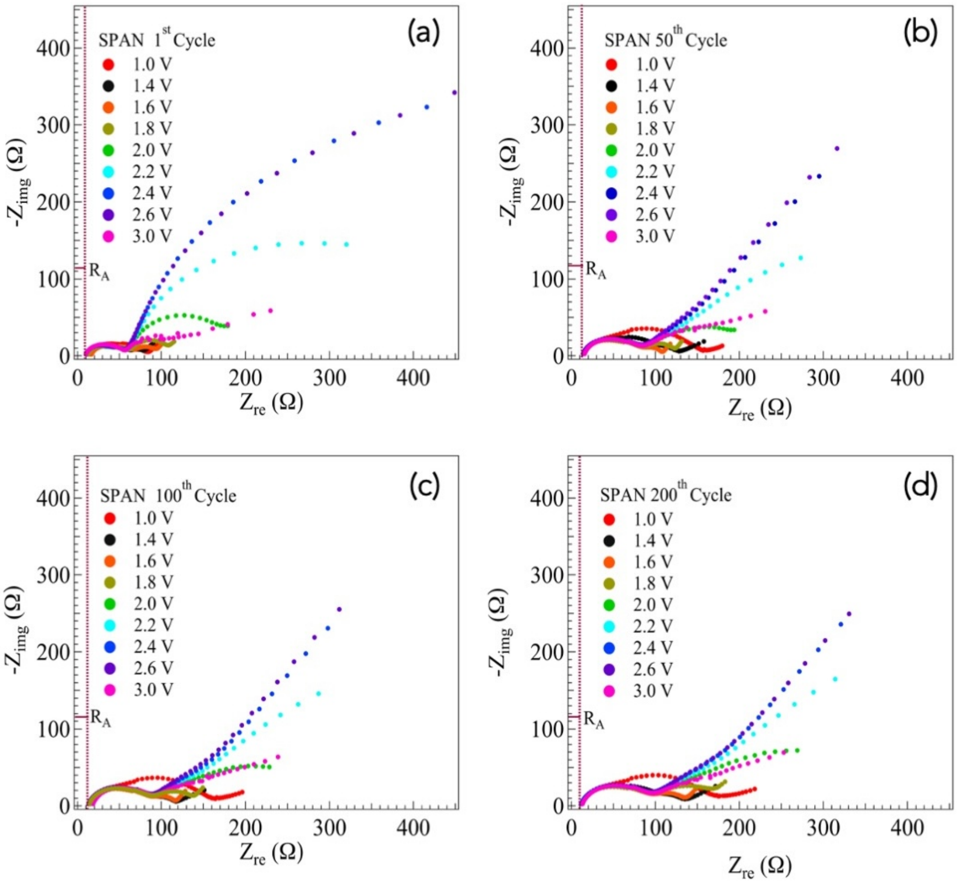

Figure S6: Nyquist plots of SPAN-35 electrodes recorded at multiple potentials (1.0–3.0 V) and different cycle numbers: (a) 1st cycle, (b) 50th cycle, (c) 100th cycle, and (d) 200th cycle. These plots show the same data as Fig. S5, except the datasets are grouped by cycle number in each panel, whereas the grouping in Fig. S5 was based on voltage.

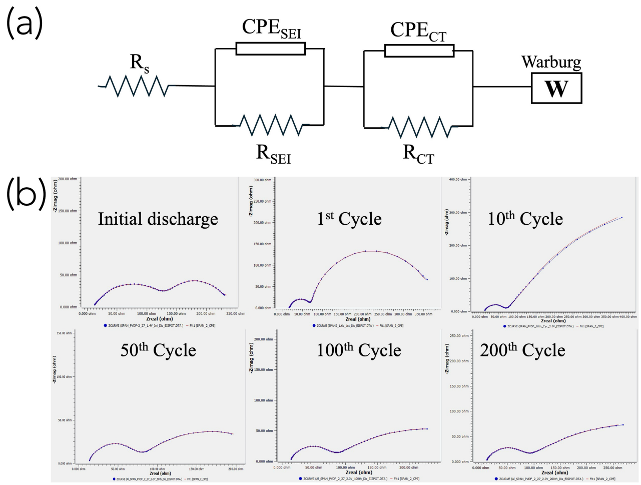

Figure S7: (a) Equivalent circuit model used for fitting the EIS data shown in Figures S5 and S6. In circuit, R_s, R_{SEI}, and R_{CT} represent the series resistance, the solid electrolyte interphase (SEI) layer, and charge transfer (CT). CPE_{SEI} and CPE_{CT} denote constant phase elements, while W is the Warburg diffusion element. (b) Representative EIS spectra with corresponding model fits at various electrochemical cycling stages. All fit parameters are listed in Table S1.

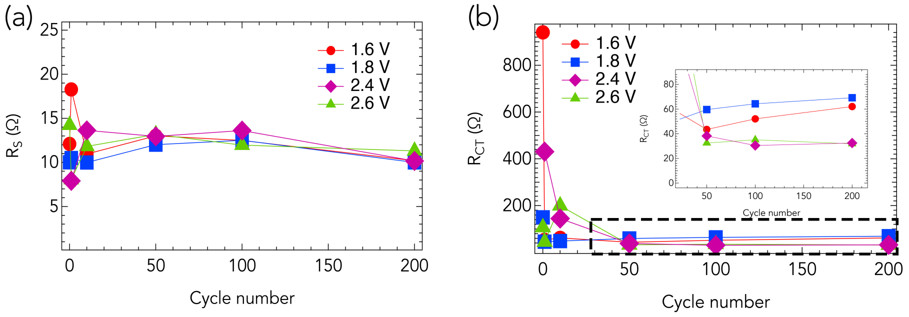

Figure S8: (a) The series resistance R_s of SPAN-35, derived from fits to Figs. S5 and S6, remain unchanged over the cycling. (b) The charge transfer resistance R_{CT} of SPAN-35 decreases significantly following the initial cycle. Upon extended cycling, R_{CT} is stabilized with little variation after 50 cycles, as shown in the inset)

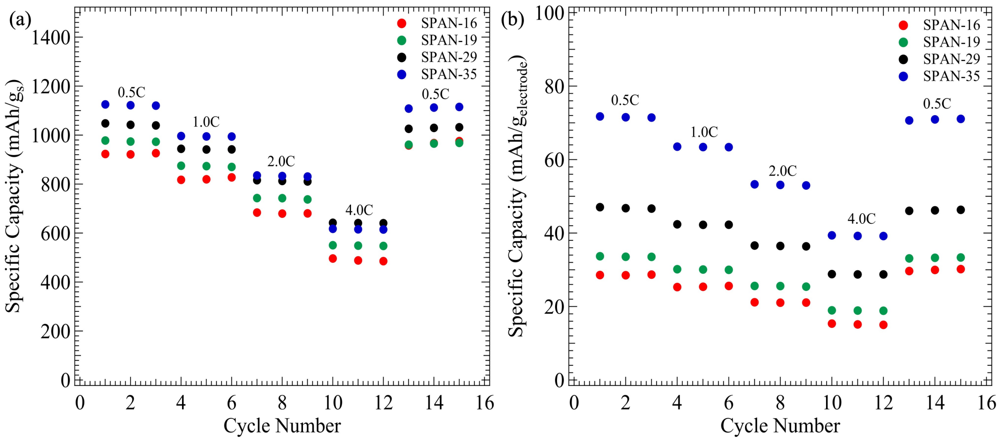

Figure S9: The rate capability profiles for SPAN cathodes with varying sulfur content showing specific capacity *vs.* cycle number of SPAN cathodes at C/2, 1C, 2C, and 4C rates (1C = 1675 mA/g) at (a) sulfur level (b) electrode level.

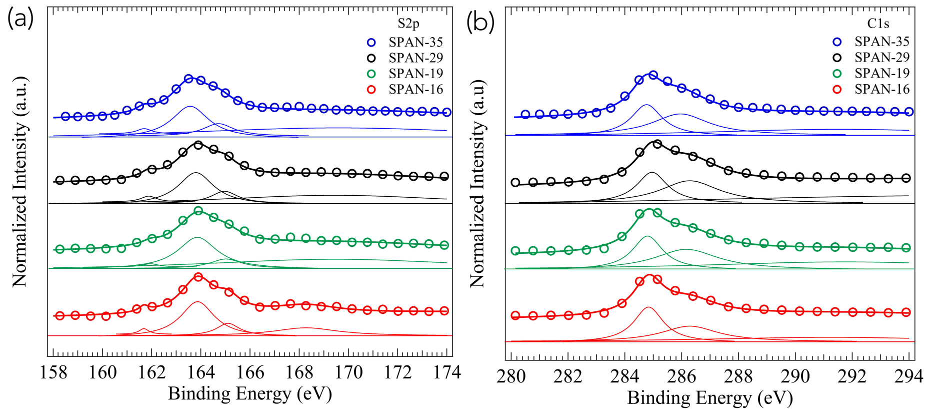

Figure S10: X-ray photoemission spectroscopy (XPS) analysis of SPAN powder samples with varying sulfur content: SPAN-16, SPAN-19, SPAN-29, SPAN-35.(a)S 2*p* and (b) C 1*s*. A broad peak is observed for all samples at 168.5 eV indicating the presence of S–O functionalities within SPAN.

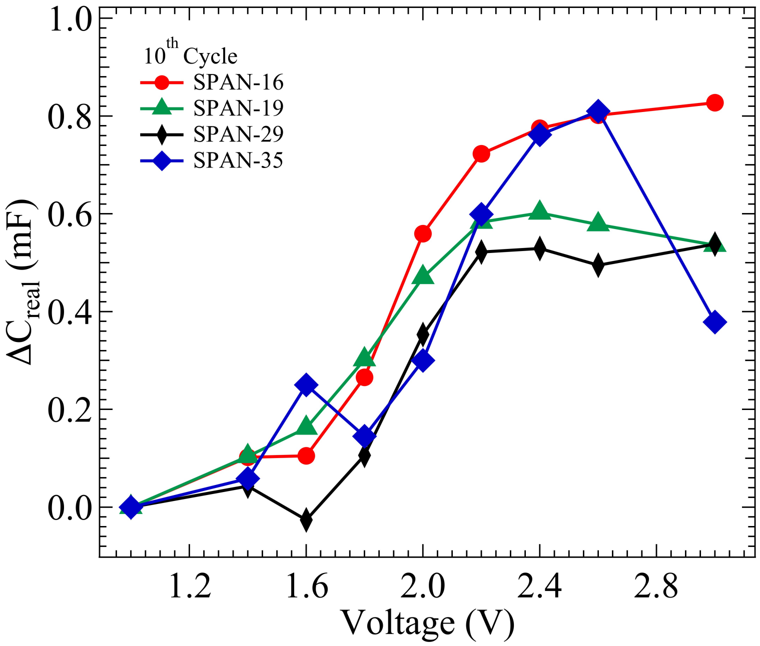

Figure S11: The changes in redox density of states ($g_r(\mu)$) for different SPAN samples measures through ΔC_{real} as a function of voltage.

Table S1: EIS fitting parameters for intial discharge, 1st, 10th, 50th, 100th, and 200th cycles at different voltages

	**Voltage (V)**	**R_s (ohm)**	**R_{SEI} (ohm)**	**R_{CT} (ohm)**	**CPE_{SEI} (S*s^a1)**	**a1**	**CPE_{CT} (S*s^a2)**	**a2**	**W (S*s^(1/2)**
	1.4	10.4554	126.4019	89.7513	5.74E-05	0.6336	1.46E-03	0.8424	0.0849
Initial Discharge	1.6	12.0976	127.9686	940.7246	4.71E-05	0.6603	5.09E-03	0.4308	2.37E-02
	1.8	10.0169	111.4785	150.0063	5.57E-05	0.6332	2.05E-02	1	1.35E-02
	2.6	14.2361	12.41	106.7477	7.77E-04	1	3.31E-05	0.6844	4.39E-03
1st Cycle	1	14.7121	43.135	23.813	1.68E-04	0.6602	5.07E-06	0.875	4.82E-02
	1.4	10.435	18.0879	46.6671	5.07E-06	0.8961	6.56E-04	0.4999	0.0548
	1.6	18.282	285.2302	53.5177	1.08E-03	0.9285	3.49E-05	0.788	0.0663
	1.8	10.4975	54.8499	46.0501	3.25E-03	0.6605	3.57E-05	0.6694	0.0906
	2	7.4494	113.4762	49.6185	2.52E-03	0.835	4.14E-05	0.6533	4.62E-02
	2.2	7.6784	46.8792	169.3414	3.14E-05	0.6781	3.27E-03	0.9908	9.26E-03
	2.4	7.9085	45.4587	430.6554	2.51E-05	0.6989	2.94E-03	0.9624	8.50E-03
	2.6	8.0596	478.5339	44.8845	2.60E-03	0.9312	2.12E-05	0.7134	8.71E-03
	3	8.9407	46.4173	96.5633	1.06E-02	0.4378	4.38E-05	0.6504	4.248
10th Cycle	1	11.3611	95.3556	28.2349	9.99E-05	0.6567	8.27E-06	0.8402	0.0519
	1.4	14.1016	49.03	32.9649	8.05E-04	0.5022	5.43E-06	0.8562	0.0702
	1.6	10.9535	39.0484	62.1267	8.22E-05	0.7594	1.80E-03	0.8891	0.0506
	1.8	9.9963	55.9029	48.4682	2.61E-03	0.533	8.89E-06	0.7909	0.1503
	2	11.2229	92.6545	55.7496	2.52E-03	0.6553	1.24E-05	0.7566	0.1675
	2.2	13.2185	285.4909	56.9439	3.14E-03	0.6147	1.34E-05	0.7492	2.7552
	2.4	13.6363	56.0934	145.0204	1.28E-05	0.7542	6.49E-03	0.8298	5.82E-03
	2.6	11.8263	53.7134	199.6503	9.87E-06	0.7785	6.85E-03	0.9423	4.61E-03
	3	11.216	46.2409	234.4442	7.05E-06	0.814	4.47E-03	0.4395	27.499
50th Cycle	1	13.6319	126.9773	14.9618	8.33E-05	0.6285	1.88E-06	0.9995	0.0532
	1.4	13.0801	85.1509	26.3616	2.07E-04	0.5674	3.79E-06	0.9098	4.40E-02
	1.6	13.0242	49.7117	43.4655	6.97E-04	0.5418	6.01E-06	0.831	0.0761
	1.8	12.0171	53.6552	59.6312	8.34E-06	0.7931	1.80E-03	0.5089	0.0774
	2	11.857	59.8584	193.2822	1.06E-05	0.7688	3.77E-03	0.4603	18.2703
	2.2	11.7408	59.775	2512.317	1.09E-05	0.7695	6.01E-03	0.3918	3.66E-02
	2.4	12.9578	30.5997	38.2163	3.07E-06	0.9048	7.06E-05	0.7139	4.34E-03
	2.6	13.1864	33.2029	32.4843	3.07E-06	0.895	4.55E-05	0.7813	3.88E-03
	3	13.6108	55.4484	309.3663	8.28E-06	0.7942	4.51E-03	0.3968	0.7105
100th Cycle	1	12.3428	131.8247	18.2921	9.44E-05	0.6206	2.19E-06	0.9775	3.78E-02
	1.4	12.3846	59.8619	44.3318	5.51E-04	0.5357	6.36E-06	0.8319	4.50E-02
	1.6	12.4899	50.4442	52.0919	9.08E-04	0.5457	7.05E-06	0.8105	0.061
	1.8	12.5095	62.0913	64.2859	9.37E-06	0.7779	1.81E-03	0.5519	0.0566
	2	12.4916	312.3093	61.9132	4.12E-03	0.4156	9.55E-06	0.7815	3.2851
	2.2	12.846	52.4309	61.6842	4.17E-03	0.4515	8.88E-06	0.7874	6.25E-03
	2.4	13.6212	48.8483	30.5149	5.47E-06	0.8392	4.06E-04	0.6091	4.38E-03
	2.6	11.967	39.8916	35.1246	4.10E-06	0.8731	1.25E-04	0.6775	4.00E-03
	3	16.1594	58.7471	239.9787	7.68E-06	0.8018	4.56E-03	0.382	4.11E-02
200th Cycle	1	10.0488	20.1187	144.5443	2.33E-06	0.9697	9.49E-05	0.6209	2.96E-02
	1.4	9.9683	47.2388	76.1682	6.62E-06	0.8337	4.01E-04	0.55	3.55E-02
	1.6	10.1979	61.0492	62.0791	8.46E-06	0.7935	9.82E-04	0.5478	4.60E-02
	1.8	10.0021	95.0174	69.2096	1.94E-03	0.5217	1.02E-05	0.7731	4.54E-02

Table S2: Correlation between CV peak positions and spectroscopic signatures from XPS and Raman analysis.

**CV Peak (V)**	**Proposed Redox Process**	**XPS Binding Energy (eV)**	**XPS Assignment**	**Supporting Raman Features**
1.9 (cathodic)	Initial reduction of longer S_n chains or confined S_8-like domains; formation of S_n^{2-}	163.5–164.0	Bridging S or S–S bonds in longer chains	Broad S–S stretching mode near 475 cm^{-1}; suppressed upon cycling
1.6 (cathodic)	Formation of Li_2SO_3	169.4–169.7	Li_2SO_4	Increased I_D/I_G ratio indicating defect generation; loss of S–S Raman band
1.4 → 1.2 (cathodic)	Deep lithiation forming Li_2S-like or terminal sulfur states	160.7–162.5	Formation of Li_2S; terminal S^{2-} states	Near-complete disappearance of S–S Raman peak; strong D-band enhancement

References

[1] Bing-Ang Mei, Jonathan Lau, Terri Lin, Sarah H Tolbert, Bruce S Dunn, and Laurent Pilon. Physical interpretations of electrochemical impedance spectroscopy of redox active electrodes for electrical energy storage. *The Journal of Physical Chemistry C*, 122(43):24499–24511, 2018.
